# A Concise Review on Porous Adsorbents for Benzene and Other Volatile Organic Compounds

**DOI:** 10.3390/molecules29235677

**Published:** 2024-11-30

**Authors:** Jerzy Choma, Barbara Szczęśniak, Adam Kapusta, Mietek Jaroniec

**Affiliations:** 1 Institute of Chemistry, Military University of Technology, 00-908 Warsaw, Poland; jerzy.choma@wat.edu.pl (J.C.); barbara.szczesniak@wat.edu.pl (B.S.); adam.kapusta@wat.edu.pl (A.K.); 2 Department of Chemistry and Biochemistry & Advanced Materials and Liquid Crystal Institute, Kent State University, Kent, OH 44242, USA

**Keywords:** volatile organic compounds (VOCs), adsorption of VOCs, porous sorbents

## Abstract

Emissions of volatile organic compounds (VOCs) such as benzene, toluene, xylene, styrene, hexane, tetrachloroethylene, acetone, acetaldehyde, formaldehyde, isopropanol, etc., increase dramatically with accelerated industrialization and economic growth. Most VOCs cause serious environmental pollution and threaten human health due to their toxic and carcinogenic nature. Adsorption on porous materials is considered one of the most promising technologies for VOC removal due to its cost-effectiveness, operational flexibility, and low energy consumption. This review aims to provide a comprehensive understanding of VOC adsorption on various porous adsorbents and indicate future research directions in this field. It is focused on (i) the molecular characterization of structures, polarity, and boiling points of VOCs, (ii) the adsorption mechanisms and adsorption interactions in the physical, chemical, and competitive adsorption of VOCs on adsorbents, and (iii) the favorable characteristics of materials for VOCs adsorption. Porous adsorbents that would play an important role in the removal of benzene and other VOCs are presented in detail, including carbon-based materials (activated carbons, active carbon fibers, ordered mesoporous carbons, and graphene-based materials), metal-organic frameworks, covalent organic frameworks, zeolites, and siliceous adsorbents. Finally, the challenges and prospects related to the removal of VOCs via adsorption are pointed out.

## 1. Introduction

**Figure 1:**
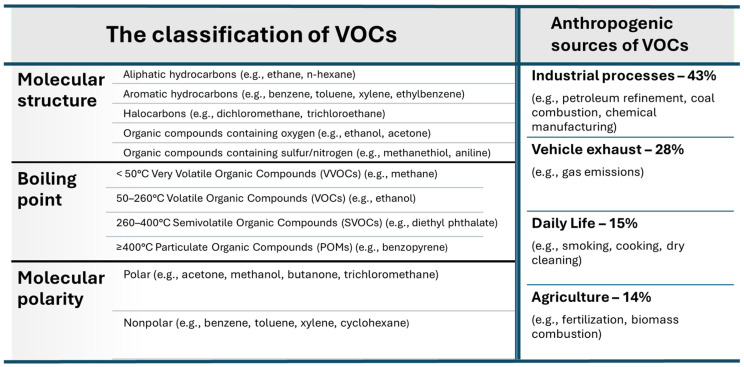
Classification of volatile organic compounds (VOCs) and different anthropogenic VOC sources in China in 2015 [4]. Adapted with permission from Ref. [4]. Copyright © 2020, Elsevier.

Volatile organic compounds that occur in air, soil, and water are organic substances with a low boiling point. According to the definition proposed by the United States Environmental Protection Agency (US EPA), a volatile organic compound is any carbon compound that takes part in photochemical reactions in the atmosphere except for carbon monoxide, carbon dioxide, carbonic acid, metallic carbides or carbonates, and ammonium carbonate. Volatile organic compounds exhibit high vapor pressure and low solubility in water. Many VOCs are man-made chemicals that are used and/or emitted in the manufacturing processes of paints, pharmaceuticals, and refrigerants. VOCs are typically industrial solvents such as trichlorethylene, fuel oxidizers such as methyl tert-butyl ether (MTBE), or by-products released during water treatment (e.g., chlorination) such as chloroform. VOCs are often components of petroleum fuels, hydraulic fluids, paint thinners, and dry cleaners. VOCs are also common groundwater contaminants. The World Health Organization (WHO) recognizes VOCs as organic compounds whose saturation vapor pressure exceeds 133,322 Pa and boiling point is between 50 and 260 °C at atmospheric pressure [1]. The WHO classifies VOCs according to their boiling points into very volatile organic compounds (VVOCs), volatile organic compounds (VOCs), semi-volatile organic compounds (SVOCs), and particulate organic compounds (POMs). Another classification of VOCs is in terms of their molecular polarity. Furthermore, VOCs can also be classified based on their molecular structure. Therefore, VOCs can be divided into the following five main groups: (1) Aliphatic hydrocarbons, which mainly come from automotive exhaust gases, asphalt, biomass combustion, petroleum processing, agricultural products, and chemical processes, e.g., n-hexane, a substance often found in workplaces and may be harmful to the human health in long-term exposure; (2) aromatic hydrocarbons, which are usually produced from coal, released during the refining of crude oil and from building materials, and occur in car exhaust gases. Among them, benzene, toluene, and ethylbenzene are typical hazardous substances due to their high toxicity and photochemical reactivity with ozone, and they are also carcinogenic; (3) halocarbons or halogenated VOCs are mainly produced in industrial processes and are found in water treatment wastes. Chlorinated compounds (Cl-VOCs) with high volatility and durability are widely present in the environment [2]; (4) organic compounds containing oxygen include alcohols, aldehydes, ketones, ethers, phenols, esters, and acidic compounds and mainly come from building materials, industrial solvents, petroleum gases, and the chemical processing of coal. Among them, aldehydes are commonly used; for instance, formaldehyde is a major indoor pollutant because it is widely used in the production of building/furniture materials and consumer goods. Acetaldehyde is a byproduct of ethanol combustion and contributes to photochemical smog. Esters and ketones come mainly from the furniture, footwear, and printing industries. Long-term exposure to esters and ketones causes severe negative effects on human health; (5) compounds containing sulfur and/or nitrogen atoms mainly originate from the chemical combustion of oils and the leather industry. Aniline and methanethiol are the two most widespread VOCs containing N and S atoms [3]. Detailed classifications with examples of representative VOCs are shown in Figure 1 [4].

Biogenic VOC emissions result from the natural circulation of VOCs. In 1995, it was estimated that the annual global flux of VOCs was 1150 Tg C (1 Tg = 10^9^ kg) and consisted of 44% isoprene, 11% monoterpenes, and 45% other VOCs [5]. Anthropogenic sources of VOCs result from human activity that, intentionally or unintentionally, increases the amount of these compounds in the environment. For instance, with accelerated urbanization and industrialization, the volume of VOC emissions from anthropogenic sources in China is expected to continuously increase above 5.9% per year (from 19.4 Tg in 2005 to 25.9 Tg in 2020) [6]. Anthropogenic sources of VOC emissions mainly come from industrial processes (43%), car exhaust fumes (28%), everyday life (15%), and agriculture (14%); see Figure 1 for more details. Industrial VOC emissions are widely related to the refining of crude oil, production and utilization of solvents, the use of fossil fuels, coal combustion, etc. Among industrial sources, coal combustion is one of the major contributors to VOC emissions (about 37%) [7].

Benzene, toluene, ethylbenzene, and xylene (BTEX) are known as the main VOCs emitted during the combustion of coal, and all have been identified as very hazardous air pollutants. Most VOCs, in particular aromatic compounds and polycyclic aromatic hydrocarbons, have an unpleasant odor and are toxic (even carcinogenic) to human health even at low concentrations (above 0.2 mg m^−3^). The commonly reported adverse effects are damage to the central nervous system and associated symptoms, kidney dysfunction, and elevated blood pressure. Some VOCs, such as ethylene and propylene, are widely used in the petrochemical industry. Although these compounds are less toxic to the human body, they are flammable and explosive. VOCs are also responsible for the greenhouse effect, especially methane, contributing to this effect over 20 times stronger than CO_2_.

A recent review showed that indoor exposure to VOCs has remained a severe public health concern in many European countries over the past decade [8]. For instance, the results identified formaldehyde and benzene as posing the highest cancer risk among the VOCs studied in France and Germany. Some countries have introduced strict regulations to control VOC emissions. For instance, US regulations request reductions in emissions of 189 pollutants by 90%, in which VOCs constitute as much as 70% of those pollutants [9].

As indicated above, VOCs are usually highly toxic, mutagenic, and often carcinogenic. Unfortunately, their concentration in the air, including indoor environments, is gradually increasing; thus, immediate treatment is needed [8]. Many technologies have been proposed to reduce VOC emissions, and they can be divided into destruction technology and recovery technology. The former technology enables the decomposition of VOCs into CO_2_, H_2_O, and non-toxic or less toxic compounds. This is accomplished by various chemical or biological methods, such as thermal/catalytic oxidation, photocatalytic oxidation, biofiltration, and plasma catalysis. Recovery technologies, such as absorption, adsorption, condensation, and separation, involve changing specific conditions in the process, e.g., temperature and pressure. Adsorption is considered one of the most promising and beneficial technologies for VOC removal because of cost-effectiveness, operational flexibility, and low energy consumption. Many porous materials have been proposed for this application, such as carbon-based materials, including activated carbons, activated carbon fibers, ordered mesoporous carbons, graphene-based materials, carbon nanotubes, and metal-organic frameworks, covalent organic frameworks, zeolites, silicas, among others. Additionally, porous composites are designed to improve VOC adsorption in terms of their capacity, hydrophobic properties, thermal stability, and regeneration capability. Apparently, the development of new cost-effective purification technologies for the removal of VOCs is still needed to protect human health and the atmospheric environment.

Interestingly, benzene is a model and representative compound in adsorption studies, especially for porous carbons. From the 1950s to the 1990s, benzene was widely used in the volume-filling theory of micropores (TVFM) by Dubinin et al. [10] to characterize the structure of porous carbons. Experimental adsorption of benzene on activated carbons was the basis for the development of important equations for adsorption theory, e.g., the Dubinin and Radushkevich, Dubinin–Astakhov, and Dubinin–Stoeckli equations [11]. In 2000, reference benzene isotherms were defined and used to determine micropore volumes and external surface areas of microporous carbons, including Carbosieve, Takeda molecular sieves, Maxsorb superactivated carbons, and an activated charcoal cloth, as well as to obtain estimates of the surface area of carbon blacks [12].

This review aims to provide a concise understanding of the main factors that govern the adsorption of VOCs on porous adsorbents. Different groups of sorbents are presented and compared, including their respective benefits and limitations. We believe that this review will inspire researchers to further explore this critical area of environmental and human health protection.

## 2. Important Characteristics of VOC in Terms of Adsorption

The physicochemical properties of adsorbate molecules, such as molecular structure, polarity, and boiling point, undoubtedly play an important role in the adsorption of VOCs on porous materials. The molecular structure of VOC molecules, i.e., their size (molecular cross-sectional area) and complexity, may determine their ability to enter pores and adsorb on the sorbent surface. The polarity of VOCs is an important factor since polar VOCs are strongly adsorbed on sorbents with polar groups, while nonpolar VOCs tend to adsorb on nonpolar surfaces. In the case of boiling point, strong adsorption interactions are observed for VOCs with high boiling points. However, their desorption is more difficult than in the case of VOCs with lower boiling points.

### 2.1. Molecular Structure of VOCs

The molecular structure of VOCs determines their ability to be adsorbed in the sorbent internal porous network because small-sized VOCs can easily reach adsorption sites with the strongest interactions, which are located in the smallest pores (micropores), while VOCs with large molecular sizes cannot enter these pores. This is known as the micropore filling mechanism and is characterized by high selectivity depending on the size and shape of VOC molecules. It includes three probable cases: (1) the size of the VOC molecule is larger than the pore size, so the adsorption process does not occur due to steric hindrance; (2) the size of the VOC molecule is equal to the pore size; thus, it can be strongly retained in the adsorbent internal structure due to the overlapping of adsorption potential of the adjacent pore walls. The adsorbed molecule is not susceptible to desorption; (3) the size of the VOC molecule is smaller than the pore size; thus, capillary condensation can easily take place in these pores, which increases the amount of adsorption. When the VOC molecular size is much smaller than the pore size, the adsorbed molecules can be desorbed. In general, the molecular size of VOCs is usually of the same order as the size of narrow micropores, except for BTEX (benzene, toluene, ethylbenzene, and xylenes) molecules. It is considered that the optimal pore size for efficient BTEX adsorption is in the range of 1.7 and 3.0 nm [4].

**Figure 2:**
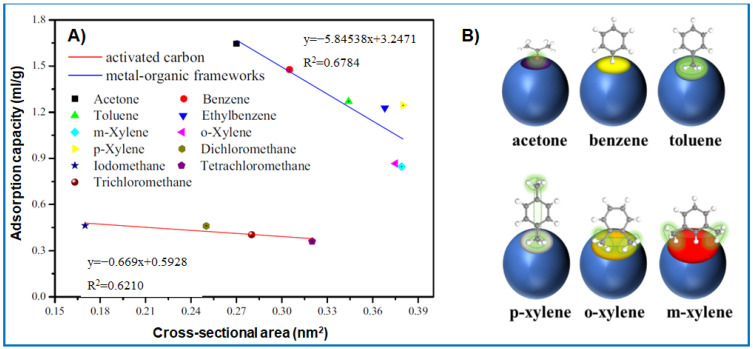
(**A**) Relationship between the VOC molecular cross-sectional area and the adsorption capacity obtained for carbons and metal-organic frameworks [15]. (**B**) Comparison of the required pore entry in sorbents for different VOC molecules such as acetone, benzene, toluene, p-xylene, o-xylene, and m-xylene [17]. Reproduced with permission from refs. [15,17]. Copyright © 2017 and 2022, Elsevier.

In general, it can be stated that the efficiency of VOC adsorption on porous sorbents, e.g., activated carbons (AC) and metal-organic frameworks (MOFs), decreases with their increasing molecular size (Figure 2A) [13,14,15]. The molecular size refers to the cross-sectional area of the molecule and differs from the minimum dimensions, such as the width, thickness, or length of the molecule [15]. For instance, the adsorption of naphthalene, having a kinetic diameter of 0.62 nm, on microporous AC was hindered due to blocking the entry of its narrow micropores [16]. In another experimental work [17], the adsorption of benzene, toluene, o-xylene, p-xylene, m-xylene, and acetone was examined on UiO-67 MOF. The surface chemistry of UiO-67 was modified with benzoic acid to increase adsorbent–adsorbate interactions. The results showed that the adsorption efficiency was related to the polarizability of the VOCs studied, the way they diffused into pores, and, above all, their molecular size. For instance, acetone molecules can easily diffuse into the smallest micropores, while benzene molecules can diffuse into slightly larger ones; meanwhile, m-xylene molecules can only penetrate the largest micropores (Figure 2B). For instance, a p-xylene molecule with two methyl groups connected to benzene ring at an angle of 180° can penetrate smaller pores compared to both o-xylene and m-xylene (Figure 2B). Finally, through desorption, density functional theory (DFT), and kinetics experiments, it was verified that the binding energy also plays an important role in adsorption and diffusion. Methyl groups interact with active sites of the adsorbent surface differently than benzene rings [18]. In this paper, it was noticed that the adsorption efficiency of xylene isomers on modified UiO-67 was also related to their polarizability [17]. It was evidenced that the greater the polarizability of the VOC molecule is, the stronger the intermolecular interactions and, hence, the higher the adsorption capacity on the MOF.

### 2.2. Boiling Point

Volatile organic compounds consist of many organic compounds with a boiling point in the range of 50–260 °C at atmospheric pressure and a saturation vapor pressure above 133.32 Pa at room temperature. The group of these VOCs includes alkanes, aromatic hydrocarbons, halogenated hydrocarbons, alcohols, aldehydes, ketones, olefins, and ethers, among others (Table 1) [19].

The process of physical adsorption of vapors on porous adsorbents is analogous to a vapor–liquid phase transition. Liquid condensation plays an important role in the overall adsorption of VOCs on porous sorbents. Adsorbates with higher boiling points exhibit stronger intermolecular interactions and are better adsorbed on porous sorbents than those with lower boiling points. Therefore, high-boiling-point compounds may displace low-boiling-point compounds in the competitive adsorption. Boiling points of VOCs should be considered as an important factor in the adsorption process. In the case of VOCs with similar boiling points, other factors influence the adsorption process, including adsorption dynamics.

Giraudet et al. [20] compared the adsorption of dichloromethane, ethanethiol, and siloxane D4 (with boiling points of 313.2, 308.2, and 448.9 °C, respectively) on activated carbon fiber cloth (ACFC). They determined that the adsorption of low-boiling dichloromethane and ethanethiol was lower (0.21 and 0.8 mmol g^−1^, respectively) compared to that of siloxane D4 (1.23 mmol g^−1^). Elsewhere [21], similar observations were reported for n-hexane and 2-propanol adsorption with boiling points of 69 and 82 °C, respectively. It was revealed that lower values of saturation adsorption capacity of the adsorbent fixed bed were obtained for compound with greater volatility and a lower boiling point (n-hexane). The compound with a higher boiling point (2-propanol) tended to be efficiently adsorbed on an activated carbon surface in a saturated state. Furthermore, Oh et al. [22] examined the adsorption of seven VOCs (methanol, ethanol, methyl ethyl ketone, benzene, n-propanol, toluene, and o-xylene) on granulated AC and established an empirical relationship between the adsorption capacity and the boiling point of VOCs, with R^2^ equal to 0.988. Except for carbonaceous sorbents, a similar effect of boiling point on adsorption of VOCs (acetone, toluene, ethylbenzene, o-xylene, m-xylene, and p-xylene) was observed for porous clay heterostructures [23]. Due to the strong affinity between high-boiling-point VOCs and adsorbents, they will easily replace lower-boiling-point VOCs during a competitive adsorption process. Wang et al. [24] examined the adsorption of eight VOCs with the following boiling points (n-butanol: 118 °C, n-butyl acetate: 126 °C, 2-heptanone: 151 °C, 1,2,4-trimethylbenzene: 170 °C, 2-butoxyethanol: 171 °C, n-decane: 174 °C, indan: 176 °C, and 2,2-dimethylpropylbenzene: 186 °C) on AC and concluded that the boiling point of the adsorbate was the main factor affecting adsorption dynamics.

The adsorption efficiency of high-boiling-point VOCs is higher due to their stronger affinity to the adsorbent; therefore, their desorption is more difficult than that of low-boiling-point VOCs. Giraudet et al. [20] compared the desorption of the selected VOCs at 147 °C and found that all adsorbed VOCs with lower boiling points, such as toluene, dichloromethane, and isopropanol, were completely desorbed, while the compound with a high boiling point, i.e., D4 siloxane, was only partially desorbed under the same conditions. Lashaki et al. [16] also reported that a higher desorption temperature is needed to remove high-boiling-point VOCs from the AC surface.

### 2.3. Molecular Polarity

The molecular polarity of VOCs directly affects their adsorption, especially on carbonaceous sorbents. In general, polar VOCs are strongly adsorbed on adsorbents with polar groups, while nonpolar VOCs tend to adsorb on nonpolar surfaces. For instance, the surface of activated carbons is typically nonpolar, with slightly polar oxygen functional groups and some inorganic impurities. This dominant nonpolar nature makes ACs not only well-suited for moist gas/vapor mixtures, but they can also selectively adsorb nonpolar or weakly polar VOCs [25]. Bansode et al. [26] compared the adsorption of various VOCs, such as bromo-dichloromethane, benzene, carbon tetrachloride, 1,1,1-trichloromethane, chloroform, and 1,1-dichloromethane, on commercially available carbons (such as Filtrasorb 200, Calgon GRC-20, and Waterlinks 206C AW). The results showed that nonpolar VOCs, C_6_H_6_ and CCl_4_, having zero dipole moment, were better adsorbed on biochar and activated carbons than polar VOCs. Furthermore, Tsai et al. [27] compared the adsorption of weakly polar CHCl_3_ on ACs and found that the adsorption capacity of ACs containing nonpolar C=C groups was 146 mg g^−1^, which was higher than in the case of ACs with polar lactone groups (74 mg g^−1^). Elsewhere, the adsorption of three compounds, acetaldehyde, benzene, and toluene, was measured on ACF [28,29]. The adsorption of polar acetaldehyde molecules was 3.2 wt%, which was significantly lower than that of nonpolar benzene (31 wt%) and toluene (53 wt%). Modification of the ACF surface with HNO_3_ enhanced the adsorption capacity of acetaldehyde up to 9.9 wt% [30]. The adsorption of polar VOCs, such as methanol and ethanol, on carbons can be enhanced to some extent by proper surface modifications [31].

Some carbon materials “naturally” exhibit relatively high polarity or after additional modifications, such as oxidation with nitric acid or ozone. Due to dipole–dipole interactions between adsorbent surface and polar VOCs, the intermolecular potential energy may decrease, thus facilitating adsorption. Qian et al. [13] compared the adsorption of CH_2_Cl_2_, CH_3_I, CHCl_3_, and CCl_4_ (with a dipole moment of 1.8, 1.59, 1.1, and 0 Debyes, respectively) on activated carbon microspheres (ACMs). They found that the adsorption of polar VOCs (CH_2_Cl_2_ and CH_3_I) on ACMs is higher than that of weakly polar and nonpolar VOCs (CHCl_3_ and CCl_4_). The sorbent contained oxygen functional groups, mainly carboxyl and carbonyl groups. Similar observations were reported analyzing the adsorption of a mixture of nine VOCs on activated carbon modified with nitric acid [32]. The above examples imply that the polarity of VOCs significantly affects their adsorption on a given sorbent; VOCs adsorb better on adsorbents with similar polarity.

## 3. Mechanisms of Adsorbate–Adsorbent Interactions

The mechanisms of interactions between adsorbate molecules and adsorbent surface play a key role in the adsorption of VOCs. Therefore, the physical, chemical, and competitive adsorption of VOCs is discussed below.

### 3.1. Physical Adsorption

Physical adsorption or physisorption is the predominant mechanism for adsorption from the gas phase on porous solids. It is the result of intermolecular interactions caused by Van der Waals forces or dispersion forces between adsorbate molecules and the sorbent surface. Due to the weak nature of these interactions, i.e., without chemical bonding and low heat of adsorption, porous sorbents can be easily regenerated, maintaining unchanged original structure [33]. The physical adsorption process can be divided into three stages. The first stage involves the transport of mass from the gas phase to the external surface of the adsorbent; then, in the second stage, diffusion of adsorbate molecules occurs into the porous structure of the solid. Finally, in the third stage, adsorption takes place; namely, adsorbate molecules settle up on the adsorbent surface [34]. The amount of adsorption depends on the porosity and surface properties of the adsorbent. VOC molecules penetrate the internal porous surface of the adsorbent by diffusion. The phenomenon of diffusion inside pores may be a combination of three mechanisms: molecular, Knudsen, and surface diffusion. The final phase of adsorption equilibrium depends on the internal porosity of the sorbent, i.e., the contribution of micro-, meso-, and macropores [15]. Macropores usually make a very small contribution to the total surface area (about 5%), while micro- and mesopores account for the largest share (95%). In mesopores, VOC molecules can also undergo capillary condensation upon certain vapor pressure, thus significantly enhancing the overall adsorption capacity of mesoporous sorbents [35].

Micropores usually constitute the main adsorption sites for VOCs adsorption, but the role of mesopores and macropores is also important in the process. For instance, too small micropores, e.g., ultramicropores (<0.7 nm), may hinder diffusion rate and significantly lower adsorption kinetics [15,33]. Physical adsorption kinetics are also related to the concentration of VOCs [34]. In most cases, only macropores are directly connected to the external surface of porous adsorbents. Mesopores are branches of macropores (similar to the vascular tissue of the human body) that create transport channels for VOC molecules to enter micropores. From the macroscopic point of view, physical adsorption on porous materials is determined by their specific surface area (SSA), pore structure, surface properties, and adsorbate properties. A high SSA and a well-developed pore structure, especially in micropores, are highly beneficial for physical adsorption. When it comes to the case of micropores, it can be stated that physical adsorption is driven by the micropore filling mechanism, capillary condensation in the case of mesopores, and larger micropores and van der Waals forces. Physical adsorption is a reversible process, and adsorbents can be easily regenerated.

### 3.2. Chemical Adsorption

Chemical adsorption or chemisorption is based on the chemical reaction that takes place between the surface (functional groups) of the adsorbent and adsorbate molecules. The difference between physical and chemical adsorption can be summarized as follows: (1) chemical adsorption usually involves single-layer adsorption, while physical adsorption involves the formation of a multilayer, especially at high pressure [33]; (2) chemical adsorption is more selective than physical adsorption because the chemical reaction occurs between specific functionalities of the adsorbent surface and certain parts of the VOC molecule; (3) the heat of adsorption caused by breaking old bonds and creating new ones during the chemical adsorption is much higher, which requires high enough activation energy. Thus, the rate of chemisorption can be accelerated at high temperatures, while low temperatures are beneficial for physical adsorption (exothermic process); (4) chemical adsorption is often irreversible and requires “advanced” desorption conditions. Therefore, the adsorbent features may change during the desorption process [36].

Usually, the chemical adsorption process involves not only chemical bonds but also physical adsorption simultaneously. Reactive sites for chemical adsorption also originate from defects of the adsorbent structure, which are, e.g., in the form of unsaturated atoms at the edges of the basal planes [37]. Unsaturated atoms also provide anchoring sites to heteroatoms, such as oxygen, hydrogen, sulfur, nitrogen, halogens, and metal cations. The surface chemistry of porous adsorbents depends on the nature of the precursors used and the synthesis procedure, which often involves chemical impregnation, heat treatment, activation, or other modifications [38]. Among the common functional groups on the adsorbent surface, groups containing oxygen and nitrogen are considered the most important for chemical adsorption [4].

Oxygen-containing groups may enhance the adsorption of polar VOCs such as methanol, ethanol, and acetone by forming hydrogen bonds. Carboxyl and hydroxyl groups have been shown to provide reactive sites for substitution or acid-base neutralization reactions. Quinone groups participate in redox reactions, oxidizing or reducing organic compounds and then generating reactive oxygen species for further oxidation [39]. Nitrogen-containing groups can be formed by post-synthesis treatment with ammonia, nitric acid, and other nitrogen compounds. These groups tend to increase the pH of adsorbents [40]. Nitrogen functional groups increase the number of active sites on the adsorbent surface, thereby improving chemical adsorption. The adsorption efficiency of adsorbents containing various nitrogen groups can be sufficiently enhanced owing to the possible high dispersion of these groups in small pores. The additional p-electrons of pyrrole and quaternary nitrogen facilitate oxidation reactions, forming superoxide ions, which are highly hydrophilic [41].

**Figure 3:**
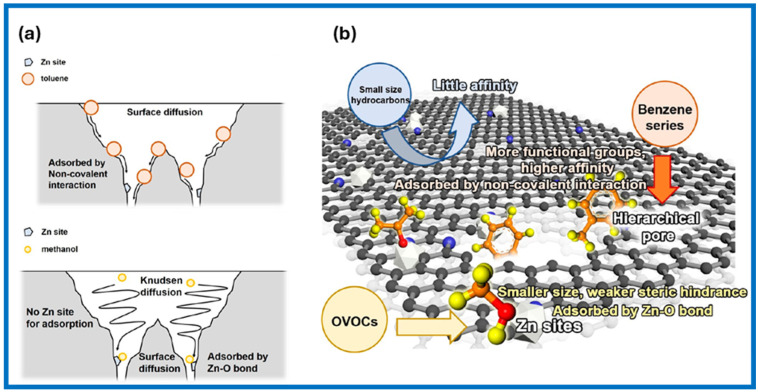
(**a**) Mass transfer process of toluene and methanol in the Zn-containing carbon sorbent (Zn-GC), and (**b**) schematic illustration of adsorption mechanism on Zn-GC for different VOCs. Adapted with permission from ref. [42]. Copyright © 2023, American Chemical Society.

Qin et al. [42] performed parallel adsorption measurements of different VOCs on MOF-derived carbon-containing Zn species (Zn-GC). They also determined the interaction between VOCs and the adsorbent surface by the difference between the maximum VOC desorption temperature and its boiling point. The results showed that adsorption of small-sized hydrocarbons, e.g., methane and propylene, on Zn-GC occurs as physical adsorption, whereas Zn species are the main adsorption sites for oxygen-containing VOCs (OVOCs), such as methanol and acetone. In the case of the benzene series of VOCs (e.g., benzene and p-xylene), the pore structure of the adsorbent mostly influences their adsorption, and the observed effects are as follows: (1) more developed hierarchical pore structure facilitates the diffusion of molecules from the benzene series into the pores and provide more space for their accommodation, and (2) molecules of the benzene series anchor to the sorbent surface via noncovalent interactions with functional groups and a hierarchical pore structure, which are the main factors for the improved adsorption affinity toward aromatic VOCs. Panels a and b in Figure 3 show the mass transfer process of toluene and methanol in the Zn-containing carbon sorbent (Zn-GC) and a schematic illustration of the adsorption mechanisms of different VOCs on Zn-GC, respectively.

### 3.3. Competitive Adsorption

**Figure 4:**
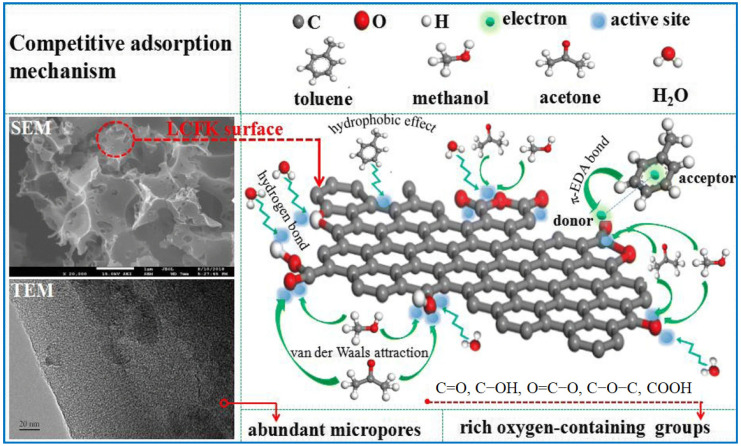
The competitive adsorption process and the relevant mechanism. LCFK: lignin-based activated carbon fibers [4]. Reproduced with permission from ref. [4]. Copyright © 2020, Elsevier.

The adsorption of two- or multi-component VOC mixtures on porous adsorbents is more complicated [43,44]. If the adsorbates show some similarities, e.g., similar interactions and cross-section area, selective separation may not be observed; they are adsorbed in a similar manner [45]. Industrial waste gases consist of at least two components: competitive adsorption is often desirable and may occur due to the different affinity of each component to the adsorbent surface. The physical and chemical properties of each component from the mixture may differentiate their adsorption affinity. The competitive adsorption is controlled by the polarity, molecular weight, boiling point, and concentration of VOCs [46]. The adsorption process is a dynamic equilibrium process of continuous adsorption and desorption. When the concentration of VOCs with a high adsorption affinity reaches a certain level, they bond on the adsorption sites and may replace those with a lower adsorption affinity. An interesting observation was obtained by analyzing the impact of different concentrations of gases (SO_2_ and NO) on the adsorption capacity of activated carbons for their sequential and simultaneous adsorption in a fixed-bed reactor [47]. The results showed that higher NO concentrations (C_NO_) increased SO_2_ adsorption, especially for C_NO_ > 200 ppm. However, higher SO_2_ concentrations (C_SO2_ > 700 ppm) limited NO adsorption, implying distinct competitive adsorption. Meng et al. [48] described the competitive adsorption process and related adsorption mechanisms for toluene, methanol, acetone, and water on active carbon fibers (ACF) (Figure 4). They showed that in multicomponent adsorption, methanol and acetone are physically adsorbed mainly through dipole–dipole interactions. At the same time, toluene molecules are adsorbed via combined physical and chemical processes because of the strong affinity between the adsorbate molecules and the adsorbent surface. The aromatic ring in toluene served as an electron acceptor, forming a stable electron donor–acceptor complex in which the main electron donors were carbonyl and lactone groups on the sorbent surface. In the competitive adsorption process, molecules of toluene or acetone may displace the adsorbed methanol molecules. Elsewhere [49], it was demonstrated that the adsorption rate of a compound with a lower molecular weight (e.g., MEK, methyl ethyl ketone) was higher than that of those with higher molecular weights (n-hexane and toluene), and then the heavier compound displaced the adsorbed lighter compound. Wang et al. [24] determined the adsorption of eight VOCs on beaded activated carbon and measured breakthrough curves for VOCs with low boiling points (n-butanol, n-butyl, and acetone). It was found that compounds with a low boiling point were desorbed because they were displaced by compounds with a higher boiling point (indan, 2,2-dimethylpropylbenzene), which exhibited stronger adsorbate–adsorbent interactions. Compounds with higher boiling points are more strongly adsorbed in porous carbons and, hence, displace the previously adsorbed molecules with lower boiling points. In the case of VOCs having similar boiling points, other factors, such as their molecular size, polarity, and the presence of functional groups and heteroatoms, determine the priority of adsorption in multicomponent systems.

Yao et al. [43] demonstrated calculations of the adsorption process of a multicomponent mixture of acetaldehyde, acetone, and ethyl acetate on AC and divided it into five stages to explain the adsorption mechanism. The priority of adsorption in the first stage of adsorption depends on the diffusion coefficients of the components in the VOC mixture. The calculated diffusion coefficients of acetaldehyde, acetone, and ethyl acetate at 25 °C are 8.19 × 10^−5^, 3.55 × 10^−5^, and 2.01 × 10^−5^ cm^2^ s^−1^, respectively. Therefore, acetaldehyde first reaches the carbon surface and is adsorbed in the active sites. Acetone and ethyl acetate are subsequently adsorbed on the surface. In the second stage, acetaldehyde molecules can first diffuse through the adsorption bed in the column, and some pre-adsorbed acetaldehyde molecules are replaced by acetone and ethyl acetate molecules. The replacement process intensifies in the third stage of adsorption, which leads to an increasing concentration of acetaldehyde outside the pores. Therefore, in this stage, its concentration in the outlet stream of the column exceeds its inlet concentration. Acetone is also detected in the outlet steam in this stage due to its diffusion through the adsorption bed. In the fourth stage, acetaldehyde can no longer be adsorbed because of the lack of free adsorption sites, and its concentration starts to be equal on both sides of the column. In this stage, the adsorbed acetone is replaced by ethyl acetate; therefore, its concentration in the outlet stream exceeds the inlet concentration. Moreover, some ethyl acetate molecules also reach the end of the adsorption column. In the fifth stage, the multicomponent adsorption mixture of VOCs on AC reaches a real equilibrium state, which is observed by equal concentrations of all components in the outlet and in the inlet of the column. To explain the replacement phenomenon, one should refer to the energy of interaction between a given adsorbate molecule and the carbon surface. Among acetaldehyde, acetone, and ethyl acetate, the latter is most strongly adsorbed on the defects-containing surface of AC. Consequently, the adsorbed amount of ethyl acetate on AC from the multicomponent VOC mixture in this study was 3.04 and 5.91 times higher than that of acetone and acetaldehyde, respectively. The results also showed that acetaldehyde and acetone desorb more easily from the AC surface than ethyl acetate, and the desorption time for acetaldehyde, acetone, and ethyl acetate was about 20, 60, and 280 min, respectively.

Competitive adsorption between water molecules and VOCs is also needed in many practical industrial processes. According to the Dubinin–Serpinsky theory, water molecules can competitively occupy adsorption sites in pores because of the reaction with surface oxygen functional groups through hydrogen bonds and capillary condensation. Liu et al. [50] observed that the adsorption of benzene without water vapor on unmodified activated carbon was 337 mg g^−1^, while at 50% and 90% relative humidity, it decreased to 256 and 166 mg g^−1^, which is a reduction of 24% and 51%, respectively. A similar observation was reported by Liu et al. [51], who examined the adsorption of isobutane on ACFC at high relative humidity. Water molecules blocked micropores of the sorbent, hindering the adsorption of hydrophobic VOCs. However, in addition to competitive adsorption, under certain specific conditions, cooperative adsorption occurs between water and hydrophilic or water-miscible VOCs. Morozov et al. [45] compared the adsorption efficiency of benzene, n-hexane, and methanol in the presence of water vapor on montmorillonites. Their adsorption capacity for hydrophobic VOCs (benzene and n-hexane) decreased significantly as the relative humidity increased from 26% to 100%. On the contrary, the adsorption of hydrophilic methanol increased as a result of its dissolution in the water film.

Overall, polar VOCs exhibit a strong adsorption affinity towards an adsorbent with a polar surface. In the competitive adsorption process, VOCs with a high boiling point and high molecular weight are privileged to occupy adsorption sites. Also, the adjacent adsorbent environment affects the adsorption of VOCs. At low temperatures, physical adsorption takes place, and the VOC uptake increases with decreasing temperature [52]. However, it should be noted that by lowering adsorption temperature, the reaction kinetics also drop. Rising adsorption temperature accelerates the diffusion of adsorbates in sorbent structure; thus, the probability of adsorption on active sites is reduced [43]. At higher temperatures, a spontaneous desorption process may occur due to the higher volatility of the adsorbate.

When adsorption is carried out outside the laboratory, the presence of water vapor in the air cannot be avoided. This factor may seriously deteriorate the adsorption process of VOCs because of the competitive adsorption of water molecules [53]. Especially, the ability to adsorb hydrophobic VOCs may be significantly reduced under high-humidity conditions. To alleviate the negative impact of water molecules, it is necessary to remove hydrophilic functional groups from the adsorbent’s surface or replace (modify) them with hydrophobic ones.

## 4. Characteristics of Adsorbents

**Figure 5:**
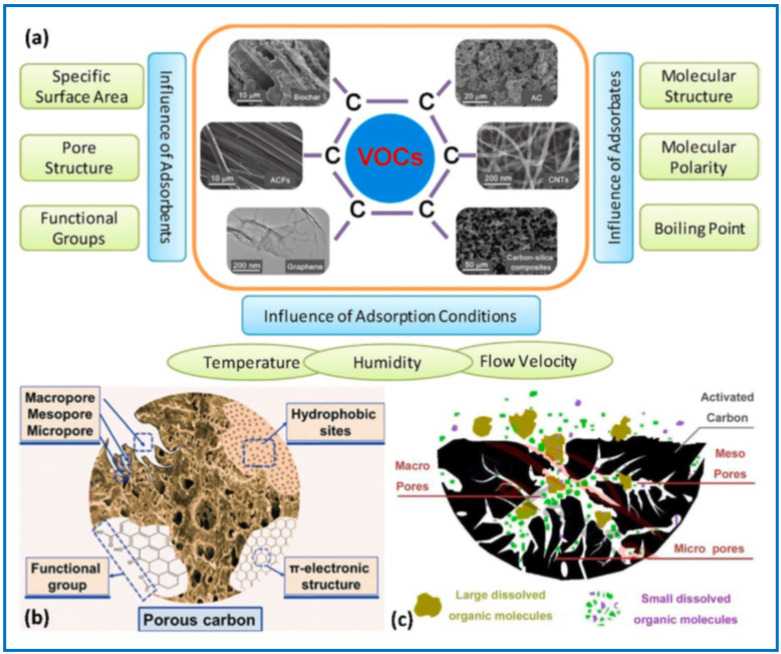
(**a**) Factors affecting VOC adsorption on porous carbons, (**b**) basic structure of porous carbons, and (**c**) VOC adsorption in different pores [54]. Reproduced with permission from ref. [54]. Copyright © 2023, Elsevier.

The above-mentioned adsorption of VOCs is influenced by the physicochemical properties of VOCs, such as the molecular structure, polarity, and boiling point, as well as the measurement conditions, such as temperature, humidity, and concentration of VOC, among others. The efficiency of the adsorption process also significantly depends on the specific features of the adsorbent used, mainly its structural parameters, such as specific surface area, pore volume, pore size distribution, as well as surface chemistry (e.g., functional groups) (Figure 5) [54].

### 4.1. Specific Surface Area and Pore Volume

The well-developed porosity is expressed by high specific surface area (SSA) and total pore volume. In general, a high SSA of material is favorable for adsorption because it is related to the presence of active sites accessible for adsorbate molecules [55]. Carter et al. [56] investigated the correlation of formaldehyde adsorption at 26 °C and the SSA of three different activated carbons: activated carbon fiber cloth (ACFC), granular activated carbon (GAC), and GAC commercially promoted for gas-phase HCHO removal. It was found that ACFC, having the highest SSA of 1084 m^2^ g^−1^ and the largest total pore volume of 0.41 cm^3^ g^−1^, showed the highest HCHO adsorption capacity of 400 mg g^−1^ compared to the two GACs. Das et al. [57] experimentally demonstrated the suitability of active carbon fibers (ACF) for the adsorption of VOCs from an inert gas stream under various operating conditions in comparison to commercially available adsorbents, such as GAC, silica gel, and zeolites. For instance, they examined the relation between the SSA and toluene breakthrough time in a fixed tubular packed bed reactor. It was demonstrated that ACF with a higher surface area (1700 m^2^ g^−1^) showed up to four times longer breakthrough time than that of ACF with a lower SSA (1000 m^2^ g^−1^). Shih et al. [58] compared the adsorption of acetone, benzene, trichlorethylene, and n-hexane on two types of multi-wall carbon nanotubes and observed a higher adsorption capacity of nanotubes with a higher SSA. It was also concluded that the morphology and structural order of carbon nanotubes affected the adsorption of organic chemicals. To enhance the value of the SSA in carbons, it is necessary to create new pores or open inaccessible pores in their internal structure. Diverse strategies of carbon modifications using thermal energy, acids, bases, microwaves, ozone, plasma, impregnation methods, etc., have been proposed to enhance their porosity [59,60,61]. More details can be found in the review [59], which covers the most important and more advanced surface treatment methods, including acid oxidation, plasma treatment, gamma radiation, treatment with rare earth elements, etc. For instance, acid treatment can effectively increase the SSA and adsorption properties of various carbon materials; however, too harsh oxidation conditions can have the opposite effect, i.e., reduce porosity due to the destruction or collapse of the existing pores. Similarly, proper alkaline treatment can also effectively increase the SSA of some carbons and, more importantly, introduce functional groups [62,63]. For instance, eucalyptus wood-derived activated carbon treated with alkaline NaOH exhibited a very high SSA of 3167 m^2^ g^−1^ and pore volume of 2.01 cm^3^ g^−1^ [63]. Yang et al. [34] investigated the adsorption behavior of ACs obtained from various raw materials, including wood, coal, and coconut shell, towards toluene at 25 °C, 200 ppm and N_2_ atmosphere. The specific surface area and total pore volume ranged from 570 to 1284 m^2^ g^−1^ and from 0.25 to 0.83 cm^3^ g^−1^, respectively. The adsorption capacity of these ACs ranged from 62.5 to 184.0 mg g^−1^. The AC with the highest porosity showed an enhanced toluene adsorption capacity. Wang et al. [64] examined the adsorption capacity of a synthesized hyper-crosslinked polymer (HCP) towards benzene at 100 °C, 550 ppm, and in an N_2_ atmosphere. The HCP was synthesized from the relatively long linker 1,4-bis(methoxymethyl)-benzene and benzyl chloride used as a monomer and had a high specific surface area of 1345 m^2^ g^−1^, large pore volume (1.75 cm^3^ g^−1^), and also hydrophobic nature, thus showing good benzene adsorption (133 mg g^−1^), which was three times higher compared to other tested adsorbents.

**Figure 6:**
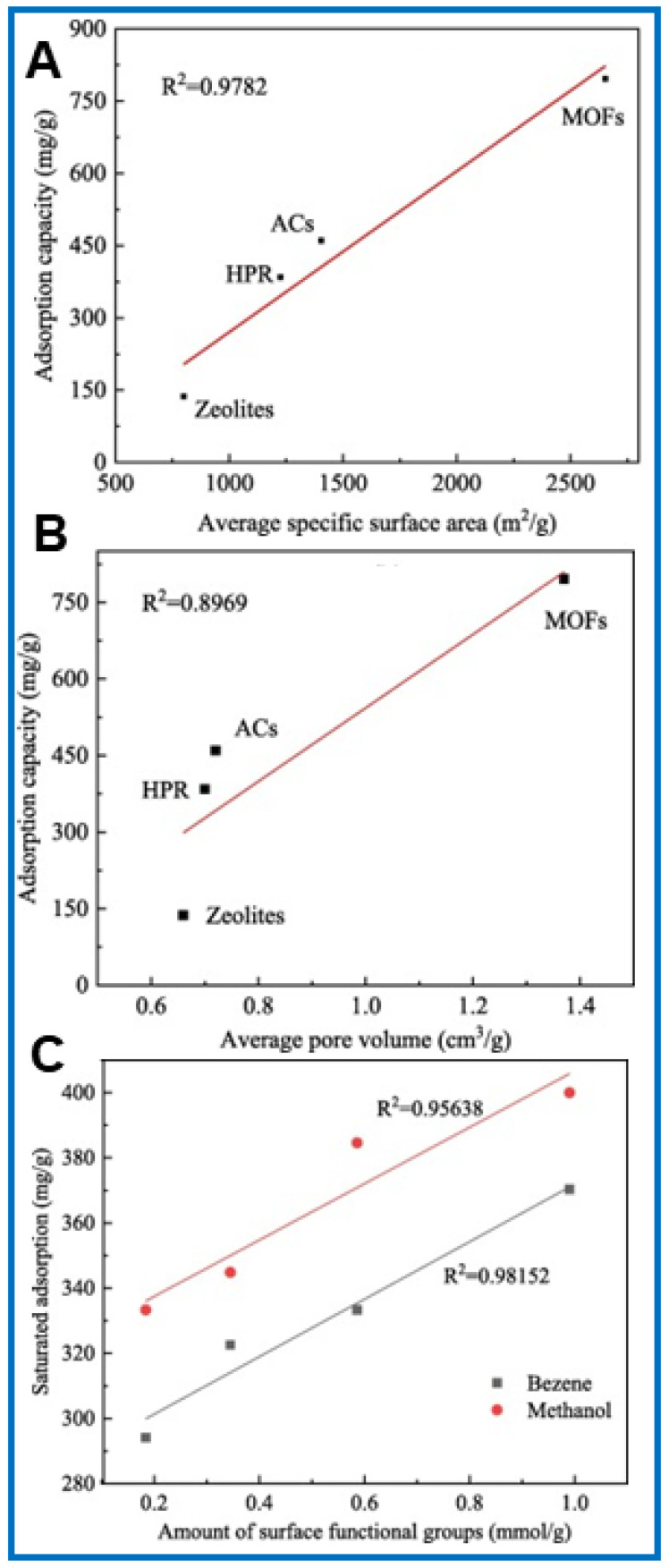
Relationship between toluene adsorption capacity and (**A**) specific surface area and (**B**) pore volume of different sorbents. (**C**) Relationship between adsorption capacity and the amount of surface functional groups [65]. Adapted with permission from ref. [65]. Copyright © 2020, Elsevier.

Li et al. [65] presented the relationship between toluene adsorption and the SSA as well as pore volume for activated carbons (ACs), zeolites, hyper-crosslinked polymer resins (HPRs), and MOFs: MOF-5, MIL-101, and MOF-177 (Figure 6A,B). These results indicate that the adsorption of toluene is linearly dependent on the value of the SSA for the examined sorbents; the correlation coefficient was as high as 0.9782. The relation of the toluene adsorption was also linear against their pore volume, and the regression coefficient (R^2^) was 0.8969. The toluene adsorption capacities of the sorbents studied were in the following order: MOFs > ACs > HPRs > zeolites. The average adsorption capacity on MOFs was 796.2 mg g^−1^, whereas the maximum adsorbed value on MOF was 1375.0 mg g^−1^, which was 1.73 times higher than that on activated carbons, 2.07 times higher than that on HPR, and 5.80 times higher than that on zeolites [65].

However, in some cases, it is not a straightforward relationship that a higher SSA guarantees better adsorption of organic compounds. For instance, this was indicated by experiments devoted to the adsorption of toluene on different AC samples [66]. An AC sample with an SSA of 798 m^2^ g^−1^ adsorbed 656 mg g^−1^ of toluene, which was almost twice a better result than that for a highly porous AC having an SSA of 2719 m^2^ g^−1^, which adsorbed 346 mg g^−1^ of toluene. A similar phenomenon was observed by Bansode et al. [26], indicating that the adsorption of bromo-dichloromethane, benzene, carbon tetrachloride, 1,1,1-trichloromethane, chloroform, and 1,1-dichloromethane on carbons is influenced by many other factors beyond the SSA or total pore volume.

### 4.2. Pore Size Distribution

**Figure 7:**
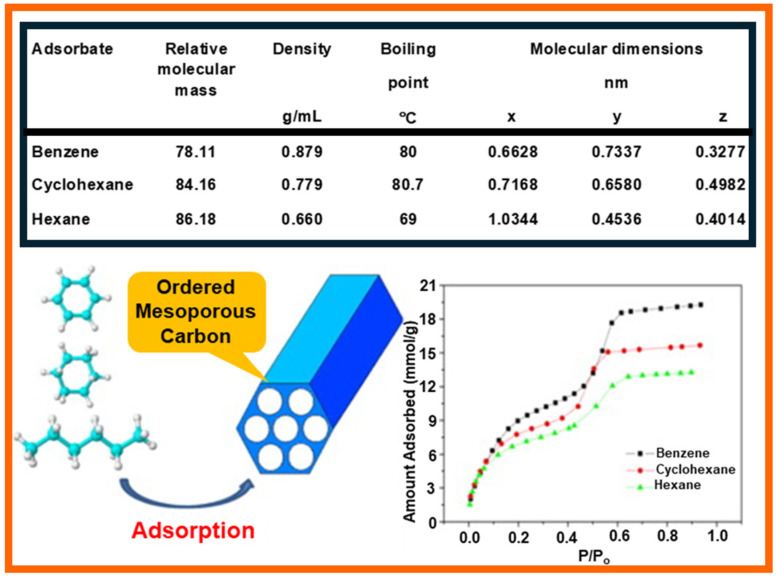
Physicochemical characterization of benzene, cyclohexane, and hexane and experimental adsorption isotherms of benzene, cyclohexane, and hexane (in mmol g^−1^) on ordered mesoporous carbon [71]. Adapted with permission from ref. [71]. Copyright © 2015, Elsevier.

Pore size distributions (PSDs) are usually calculated using density functional theory (DFT) and the Barrett–Joyner–Halenda (BJH) or Kruk–Jaroniec–Sayari (KJS) methods based on the adsorption isotherm of nitrogen, argon, or benzene [67,68]. The pore size distribution in porous sorbents may determine their adsorption capacity towards VOCs [37,62,69]. Their pores can be divided into micropores (pore size < 2 nm), mesopores (2 nm < pore size < 50 nm), and macropores (pore size > 50 nm). Generally, micropores play the main role in adsorption, while mesopores increase molecular diffusion inside particles of porous materials and, hence, shorten the adsorption time. Therefore, the adsorption of VOCs on porous materials is influenced by the pore size in different ways. It has been shown that micropores, especially small micropores with sizes smaller than (or around) 1 nm, dominate the adsorption of VOCs. For instance, it was calculated that acetone and ethyl acetate, having molecular diameters of 0.48 and 0.60 nm, respectively, can be more strongly adsorbed on carbons with a pore size of about 1.0 nm than in pores with a size of about 2.4 nm [43]. This is because the strongest Van der Waals forces between the VOC and carbon surface are provided in pores about two times larger than the diameter of adsorbate molecules. Consequently, enlarging the pore size of the sorbent has a lower effect on the adsorption of such small-sized VOCs. Pores with a size larger than 2.4 nm in AC did not affect the calculation results since their size is much larger than the molecular diameter of the examined VOCs. Qian et al. [13] compared the adsorption of chloromethanes and CH_3_I on activated carbon microspheres (ACMs) with micropore sizes ranging from 0.4 to 1.5 nm and found that the adsorption capacities (calculated in ml g^−1^) for all adsorbates are close to their volumes of micropores. Similar observations were reported in the case of toluene and acetone adsorption on pinewood chip-derived activated carbon, characterized by a large micropore volume of 0.70 cm^3^ g^−1^ (comprising 75% of the total pore volume) [70]. To determine the correlation of the pore size distribution on VOC adsorption, it is necessary to eliminate the influence of chemical functional groups and other pore structure parameters, e.g., specific surface area. It was reported that after the removal of oxygen functional groups via thermal treatment, the volume of small micropores with sizes below 0.7 nm in AC was a better controlling factor for benzene adsorption than the volume of micropores (up to 2 nm) [29]. The experiment was performed for two VOCs (benzene and toluene) at low concentrations (200 ppm). The developed microporosity in porous materials is usually highly beneficial for the adsorption of VOCs, especially for low VOC concentrations. However, the diffusion of organic compounds may be severely hindered in narrow pores, which leads to low adsorption rates, especially for higher VOC concentrations [27,55]. Tsai et al. [27] compared adsorption kinetic curves for several VOCs (chloroform, acetone, and acetonitrile) on commercial AC, two types of ACF, and sludge-derived carbon. The diffusion coefficient of VOCs on AC and sludge-derived adsorbent was ~10^−4^ cm^2^ s^−1^. The diffusion coefficient of VOCs on carbon fibers ranged from 10^−8^ to 10^−7^ cm^2^ s^−1^. Smaller pores in carbon fibers were responsible for lower values of the diffusion coefficient. Similarly, Wang et al. [71] reported that the diffusion rate constants of VOCs (benzene, cyclohexane, and hexane) in ordered mesoporous carbon (OMC) were almost twice as high as in the case of microporous AC. Figure 7 shows the experimental adsorption isotherms of benzene, cyclohexane, and hexane (along with their physicochemical characteristics) on OMC [71]. The PSD determined by the BJH method revealed bimodal pores with average pore sizes of 1.8 and 5.0 nm.

Optimal adsorption occurs when the pore size is comparable (or about twice larger) to the size of the adsorbate molecules; therefore, mesopores can be more suitable for the adsorption of large VOC molecules. Carrasco-Marín et al. [72] reached this conclusion by measuring CH_3_I adsorption on several activated carbons using gas chromatography. Koresh et al. [73] confirmed this fact based on adsorption studies of cyanogen chloride on activated carbon cloths. Kim et al. [74] reported that as the pore size decreases, the adsorption capacity of sorbents increases toward low-molecular-weight VOCs (e.g., MeOH, EtOH, and iso-propanol) and decreases for adsorption of high-molecular-weight VOCs (e.g., m-xylene and methyl ethyl ketone). Micropores facilitate adsorption of small-sized VOCs, while large-sized VOCs are better adsorbed in mesopores. It should be noted that even if the VOC has a smaller molecular size than the pore size, there is no guarantee that the VOC will penetrate these pores. Kim and Ahn [55] compared the adsorption of VOCs (benzene, toluene, o-, m-, p-xylene, methanol, ethanol, isopropanol, and methyl ethyl ketone) on zeolites, i.e., mordenite and X- or Y-type faujasite, and it was found that VOCs with small kinetic diameters of 0.38–0.53 nm can be easily adsorbed on these zeolites. Moreover, they found that the volume of mesopores in zeolites had a high impact on the overall adsorption of VOCs and can be correlated with the adsorbed amount of VOCs. Moreover, even if a small amount of VOCs is adsorbed in meso- or macropores compared to micropores, these larger pores provide the necessary transport channels for VOC molecules and are especially needed for large-sized adsorbate molecules [66]. Chiang et al. [75] studied the influence of the pore structure of activated carbons on the adsorption of four different VOCs, i.e., benzene, tetrachloride (CCl_4_), chloroform (CHCl_3_), and methylene chloride (CH_2_Cl_2_). It was found these substances are mainly physically adsorbed in micropores and mesopores. It was demonstrated that the adsorption of benzene was favored compared to other adsorbates due to its higher heat of adsorption and lower entropy change. Crespo and Yang [76] studied the adsorption of benzene, thiophene, and cyclohexane on single-wall carbon nanotubes with pore sizes of 1.2 nm and 1.68 nm and revealed that carbon nanotubes with smaller pores showed better adsorption capacities toward the VOCs studied.

Baur et al. [77] examined the influence of morphology and pore structure of ACFs on their toluene adsorption capacities based on adsorption enthalpy calculations. They found that higher adsorption strength was observed for ACF with pores below 1 nm than for ACF with supermicropores having pore sizes between 1 and 2 nm. Similarly, Lillo-Ródenas et al. [28] suggested that narrow micropores (ultramicropores) played a key role in the adsorption of benzene on ACF. Elsewhere, it was suggested that pore size distribution is the main parameter influencing the removal of VOCs at low concentrations. A narrow PSD with an average pore size between 0.8 and 1.0 nm was shown to be the most suitable for achieving high VOC adsorption [78].

Overall, narrow micropores serve as active adsorption sites for small-sized VOCs. In contrast, larger micropores and small mesopores can effectively capture larger VOC molecules. The presence of larger mesopores in the sorbent structure is also beneficial because of the facilitated molecular diffusion through the adsorbent structure and the possible condensation of VOCs at higher pressures, which may significantly contribute to the total adsorption capacity of sorbents.

### 4.3. Surface Functional Groups

Usually, the adsorption process of VOCs involves both physical and chemical interactions with the adsorbent surface. The type and amount of chemical functional groups on the surface of the adsorbent have a significant impact on the amount of VOCs adsorbed. Boehm titration can be used to analyze functional groups. For instance, carboxyl and hydroxyl groups have a strong ability to absorb electrons [65]. The adsorption of aromatic VOCs is mainly based on the formation of electron-involved complexes between acidic functional groups (e.g., carboxyl) on sorbent surface and aromatic rings [79]. The formation of hydrogen bonds between functional groups containing oxygen and benzene rings further strengthens the binding efficiency. The relationship between the adsorption of VOCs and the amount of surface functional groups is shown in Figure 6C [65]. As can be seen in this figure, the amount of benzene and methanol adsorption in the saturated state is a linear function of the number of chemical functional groups on the adsorbent surface. The correlation coefficient of methanol adsorption was 0.95638, and in the case of benzene adsorption, it was as high as 0.98152. Kim et al. [80] studied the adsorption efficiency of VOCs on amine-functionalized MOF (MIL-125-NH_2_). The results indicate that adsorption tends to follow the order of VOC polarity (p-xylene < toluene < benzene < acetone < isopropanol). This is due to the strong interactions of polar VOCs with the amine groups of MIL-125-NH_2_.

**Figure 8:**
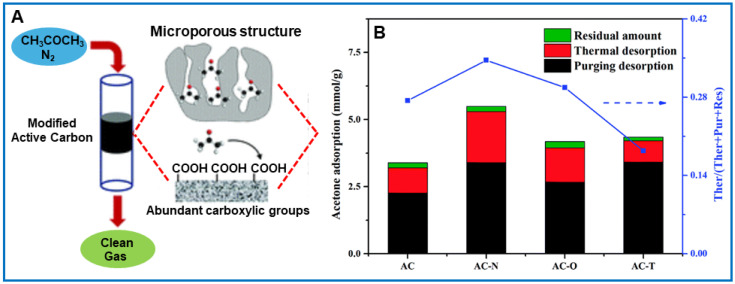
Adsorption/desorption of acetone on activated carbon. (**A**) Schematic illustration of the process. (**B**) Contribution of the purging desorption, thermal desorption, and the residual amount in ACs to the total adsorption capacity. AC: active carbon, AC-N: active carbon modified with HNO_3_, AC-O: active carbon modified with H_2_O_2_, and AC-T: heat treatment of active carbon [82]. Figure adapted with permission from ref. [82]; licensed under CC-BY-NC 3.0.

VOC adsorption and selectivity on an adsorbent can be altered by the chemical modification of its surface. For instance, basic functional groups can be incorporated by treating it with ammonia, or the amount of acidic functional groups can be increased by oxidation. Kim et al. [74] used different types of impregnation methods to modify coconut shell-derived activated carbon. The activated carbon impregnated with phosphoric acid showed improved adsorption capacity towards benzene, toluene, and xylene. Similarly, chemical modification of GACs with ammonia promoted the adsorption of hydrophobic VOCs [62]. Modifications of commercial activated carbon (NORIT) through various chemical and thermal treatments (including oxidation in gas and liquid phases) led to materials with different surface chemistry [40]. It has been shown that oxidation in the gas phase mainly increases the concentration of surface hydroxyl and carbonyl groups, while oxidation in the liquid phase mainly increases the concentration of carboxyl groups [81]. Yu et al. [82] investigated the effect of surface functional groups in AC on the adsorption of acetone at 27 °C, 500 ppm and in an N_2_ atmosphere. AC modified with nitric acid showed high acetone adsorption (5.49 mmol g^−1^). This was attributed to the increased interactions between carboxyl groups on the AC surface and acetone molecules (Figure 8). Similarly, Zhou et al. [83] observed that AC modified with magnesium oxide showed a high equilibrium amount of adsorbed acetone (432.7 mg g^−1^) at 25 °C and 85.21 g m^−3^. Apparently, the introduced oxygen functional groups acted as additional active sites that ensured a strong affinity of polar acetone molecules toward the adsorbent surface. Baur et al. [77] compared the adsorption of acetaldehyde on ACFs decorated with La_2_O_3_, CaO, MgO, ZnO, and Al_2_O_3_ and found that after modification, adsorption can be enhanced even 10 times compared to bare ACFs. The efficiency of the VOC adsorption process was significantly influenced by the physicochemical properties of the adsorbent and also the adsorption process conditions.

## 5. Summary of VOCs Adsorbents

Activated carbons, zeolites, and hyper-crosslinked polymer resins are listed by the U.S. Environmental Protection Agency (US EPA) as the three main porous groups of materials for the effective adsorption of VOCs, especially activated carbons [65]. In our opinion, metal-organic frameworks and graphene-based materials may be added to the list soon due to their potentially high SSAs and numerous chemical functional groups.

### 5.1. Activated Carbons and Biochars

The rapid development of modern society in the 20th and early 21st centuries forced the mass production of activated carbons. For example, in 2015, the world production of ACs was as high as ~12,804,000 tons [84]. AC is considered a universal adsorbent due to its high SSA (usually 500–1500 m^2^ g^−1^), large pore volume (0.5–1.5 cm^3^ g^−1^), and a high potential to effectively adsorb VOCs (10–600 mg g^−1^). The production cost ranges from USD 1000 to USD 1500 per ton [85]. It can be produced in various forms and used in environmental applications, such as wastewater treatment, water treatment, soil remediation, and air purification, including in the removal of VOCs [86]. Table 2 presents the adsorption properties of exemplary activated carbons for VOCs under specific adsorption conditions [15].

As can be seen from the data presented in Table 2, ACs can be used for the adsorption-based removal of most types of VOCs, including alkanes, alcohols, ethers, aldehydes, ketones, esters, aromatic compounds, etc. Based on this list, it can be summarized that, typically, the adsorption of VOCs on ACs is in the range from dozen to several hundred milligrams per gram of the adsorbent, depending on the physicochemical properties of the AC used, such as the specific surface area, pore volume, pore size distribution, chemical functional groups, etc., and the properties of VOCs, such as molecular size and polarity, as well as adsorption conditions such as temperature, humidity, pressure, etc. Due to the prevailing physical adsorption mechanism, carbons with high porosity are usually efficient VOC sorbents. The presence of chemical functional groups on ACs makes them also suitable for the adsorption of polar VOCs due to the possibility of chemical adsorption [87]. Acs especially afford high VOC adsorption at room temperature, low/medium concentrations, and in an N_2_ atmosphere.

Biochars are a group of carbon materials produced from biomass by slow pyrolysis in an inert atmosphere. Biochars production costs can comprise even 1/6 of the commercial price of ACs [88]. The properties of biochars mainly depend on the raw materials used as precursors and production conditions [89]. For instance, Zhang et al., in a review paper [15], assessed 15 biochars obtained from five popular raw materials for the adsorption of acetone, cyclohexane, and toluene at room temperature and VOC flow of 50 mL min^−1^. The specific surface area of these biochars ranged from 0.1 to 388 m^2^ g^−1^, and their adsorption capacity toward VOCs was less than 90 mg g^−1^. Physical or chemical activation is usually needed to develop the porosity of biomass-derived carbons and, hence, their adsorption properties [90,91]. For instance, KOH activation enabled the increase in SSA of biochar acquired from bio-wastes from 228 to 1397 m^2^ g^−1^ and pore volume from 0.02 to 0.51 cm^3^ g^−1^ and, therefore, enhanced its benzene adsorption capacity to 144 mg g^−1^ [92]. Elsewhere [89], biochar activated with 50% H_2_O(g)/50% N_2_ steam exhibited an SSA of 950 m^2^ g^−1^ and a toluene adsorption capacity of 227 mg g^−1^, comparable to that of commercial AC.

Overall, the porosity of non-activated biochars is rather low, which limits their ability to adsorb VOCs [91,93]. Significant improvements in their adsorption properties can be achieved through physical or chemical modifications (activation); thus, they are considered as a potential alternative to commercial activated carbons. For carbon sorbents, the main adsorption mechanism is based on physical adsorption. Therefore, VOCs can be relatively easily desorbed, which can cause, to some extent, secondary environmental pollution under specific conditions. On the other hand, the adsorption of some VOCs, especially in the case of the involved chemical adsorption, might cause problems with the regeneration of ACs, thus affecting their lifetime and the costs of the process [94]. Moreover, carbons are vulnerable to degradation by fire, which may pose a real risk, especially in highly exothermic adsorption processes [95].

### 5.2. Activated Carbon Fibers

**Figure 9:**
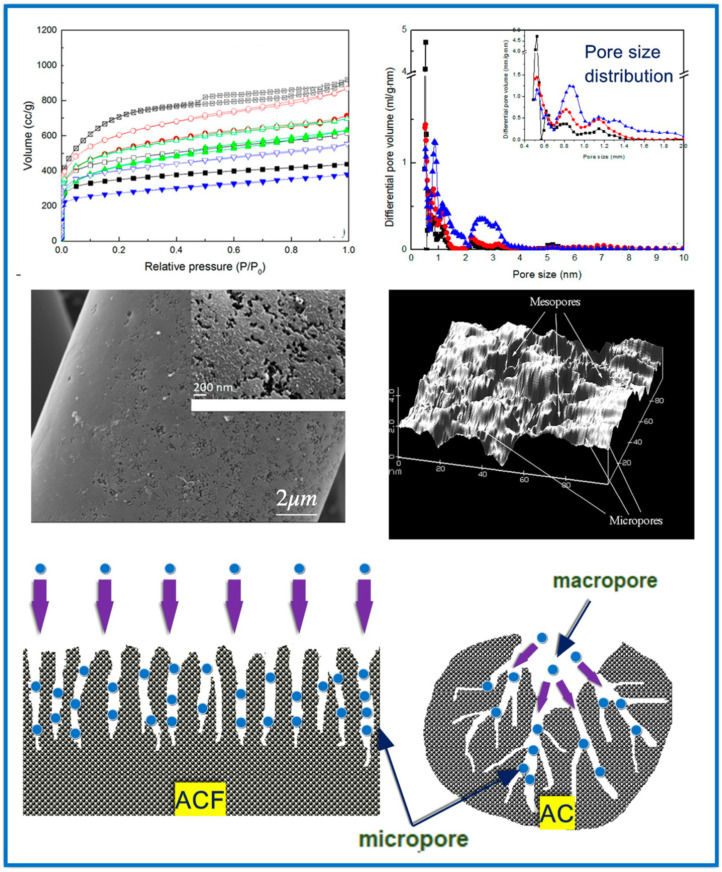
Activated carbon fibers (ACF) characteristics and VOC adsorption mechanisms [15]. Reproduced with permission from ref. [15]. Copyright © 2017, Elsevier.

Activated carbon fibers (ACFs) are fibrous carbon materials produced by the carbonization and activation of organic fibers (such as polyacrylonitrile fibers, cellulose fibers, phenolic resin fibers, pitch fibers, etc.) at a temperature of 700–1000 °C in a water vapor or carbon dioxide atmosphere [96]. ACFs may have unique morphology and properties compared to activated carbons (Figure 9). For instance, faster adsorption–desorption kinetics and higher mass transfer rates are provided by their thin-fiber shape morphology and existing short and straight micropores [97,98,99].

ACFs are typically hydrophobic and contain lower amounts of surface oxygen groups (<900 μmol g^−1^) than ACs (1000–4500 μmol g^−1^) [100]. Therefore, they may show superior adsorption properties towards nonpolar VOCs (e.g., benzene and toluene) and rather low efficiency for polar compounds (e.g., acetaldehyde and acetone) [28,29,77]. Proper surface modifications may enhance adsorption on ACFs toward polar adsorbates. The introduction of oxygen functional groups is the most commonly used modification procedure to increase the affinity between the ACF surface and polar VOCs [31,101]. For instance, the adsorption efficiency of low concentrations of ethanol at 20 °C on ACF modified with CuSO_4_ was enhanced from 480 to 560 mg g^−1^ [31]. The selection of the ACF activator (CO_2_ or H_2_O) has a significant impact on the adsorption process of VOCs. Activation with steam would be beneficial for the adsorption of polar compounds because of the additionally formed surface oxygen groups during the activation process [102]. For instance, ACFs prepared from pitch fibers activated with a mixed gas of CO_2_/H_2_O exhibited a large micropore volume of 0.435–0.715 cm^3^ g^−1^ and a high SSA of 1000–2000 m^2^ g^−1^. These ACFs showed a strong adsorption affinity toward chloroform vapors, reaching an adsorption capacity as high as 1004 mg g^−1^ at 22 °C, which is superior as compared to the commercial ACF [103]. Kang et al. [104] developed an ACFs-based material that acted as both an adsorbent and a catalytic oxidizing agent for VOC removal, thanks to the incorporated MnO_2_ catalyst on the carbon surface. Under typical adsorption conditions, the adsorption efficiency of MnO_2_-loaded ACF for toluene removal was lower compared to pure ACF, but the process was significantly improved at higher temperatures due to the catalytic oxidation of toluene, reaching 90% conversion at 280 °C.

ACFs have a high potential to be used for VOC adsorption. Except for their high adsorption capacities, they also often show better adsorption selectivity compared to activated carbons [105]. However, the practical application of ACFs in the industry is limited mainly due to the high cost of fibrous precursors and associated manufacturing costs [106,107].

### 5.3. Ordered Mesoporous Carbons

Ordered mesoporous carbons (OMCs) are another group of potentially attractive carbon-based sorbents for VOC adsorption. They are synthesized by hard or soft templating methods, usually from phenolic precursors, e.g., phenol, resorcinol, and phloroglucinol [108,109,110,111]. Their characteristic feature is the presence of ordered mesopores that can serve as transport channels for adsorbate molecules, thus facilitating diffusion. Moreover, it is considered that a large surface of mesopores can also be crucial for adsorption [71,112]. Wang et al. [71] studied the adsorption of benzene, cyclohexane, and hexane on OMC and revealed that the diffusion rate constants of VOCs on OMCs are almost two times higher than that of AC (diffusion rate of VOCs on AC is in the range of 2–4 × 10^−3^, and on OMC, about 8 × 10^−3^). Benzene adsorption on OMCs was influenced by their pore size distributions, and OMCs with narrower mesopores showed better performance in the adsorption experiments, reaching benzene adsorption capacity of 0.114 mmol g^−1^. Microporous carbons usually show superior VOC adsorption capacity in comparison to mesoporous sorbents because their narrow pores have similar sizes to that of VOC molecular size, which provide strong adsorption sites. However, the desorption process of VOC molecules from highly microporous sorbents may be significantly hindered. In contrast, the desorption process of larger molecules is facilitated by the presence of mesoporous channels in OMCs [113,114]. There are relatively not many papers devoted to the properties of OMCs for the adsorption of VOCs. However, a few research groups pointed out their high potential for adsorption of benzene, reaching high adsorption capacities of 17.3 mmol g^−1^ at 25 °C [71,115]. In the comparative study of highly acidic, sulfonated carbons, OMCs, and various commercially available ACs, the highest benzene adsorption capacity was achieved on OMC samples (14 mmol g^−1^) [115].

### 5.4. Graphene Materials

Graphene is a two-dimensional structure composed of a single layer of hexagonally arranged carbon atoms with sp^2^ hybridization [116]. Graphene-based porous materials such as graphene oxides (GOs) may have a large amount of oxygen-containing groups such as carboxyl, hydroxyl, and epoxy groups and are explored in the wastewater treatment containing heavy metals, dyes, and organic or inorganic pollutants as well as in the purification of air from, e.g., NH_3_, H_2_S, or VOCs [31,101]. Due to the large amount of oxygen functional groups, the GO surface is highly hydrophilic. Therefore, the adsorption of VOCs is influenced by the presence of water vapor. Oxygen-containing functional groups can be quite easily removed through chemical reduction (or thermal treatment), yielding reduced graphene oxide (RGO). The reduction process increases hydrophobicity and the contribution of sp^2^ hybridized carbon atoms, which is beneficial for the adsorption of VOCs (especially nonpolar) over water [98,117]. Yu et al. [118] compared the adsorption of benzene and toluene on GO and RGO at room temperature, 50 ppm, and in an N_2_ atmosphere. The SSAs of the examined GO and RGO were 236.4 and 292.6 m^2^ g^−1^, respectively. RGO showed a higher adsorption capacity for benzene and toluene (276.4 and 304.4 mg g^−1^, respectively) than GO (216.2 and 240.6 mg g^−1^, respectively), which originated from its more hydrophobic nature, lower oxygen content, and higher porosity. Szczęśniak et al. [119] synthesized a graphene-based material using a modified Hummer’s method, followed by thermal exfoliation, which exhibited a high contribution of mesopores and, to the best of our knowledge, the highest adsorption capacity towards benzene, namely 33.6 mmol g^−1^ at 20 °C and relative pressure close to unity. High benzene adsorption was possible due to enhanced π–π interactions between separated defects containing graphene layers and benzene rings.

The main limitations of using graphene and its derivatives in environmental applications are related to their expensive and/or complicated synthesis and the tendency of graphene layers to aggregate (restack). Graphene materials are often proposed to prepare functionalized composite materials [120,121,122]. A special emphasis is placed on MOF/GO composites, which are also potential candidates for effective adsorption of VOCs [123,124]. For instance, using MOF/graphene oxide composites [123,124], the adsorption of acetone (20.1 mmol g^−1^ at 15 °C) was 44.4% higher than that on bare MOF [123], and the adsorption of n-hexane (1042.1 mg g^−1^ at 25 °C) was two times higher than that on MOF [124] and 2–11 times higher than on AC [124] and zeolites [124]. The high adsorption capacity of MOF/GO composites was attributed to their high SSA up to 3500 m^2^ g^−1^, large pore volume of 1.75 cm^3^ g^−1^, and strong dispersion forces originating from the GO component, which is characterized by the dense packing of atoms [124]. Elsewhere [119], the composite consisting of mesoporous graphene and MOF with a weight ratio of 1:2 showed an almost twice-larger benzene adsorption capacity, i.e., 24.5 mmol g^−1^ at 20 °C and relative pressure close to unity, in comparison to that of pure MOF. Graphene materials can also serve as carriers of catalysts for the catalytic oxidation of VOCs. For example, MnO_2_/graphene composite can effectively catalytically decompose toluene due to the synergistic effect of graphene and MnO_2_ properties [125].

### 5.5. Metal-Organic Frameworks and Covalent Organic Frameworks

Metal-organic frameworks (MOFs) are composed of metal ions or clusters coordinated with organic ligands [126]. The structure of MOFs can be flexibly controlled by selecting appropriate organic ligands. Over the last twenty years, MOFs have attracted great interest worldwide due to their unique properties, such as ultrahigh surface area (up to 6000 m^2^ g^−1^), good thermal stability (>400 °C), adaptable pore structure, and easy functionalization [18]. MOFs are proposed for various applications, e.g., in gas storage, separation, heterogeneous catalysis, and the detection of VOCs [127]. Open metallic sites in these porous frameworks may effectively enhance the adsorption of diverse VOCs [128,129]. Vellingiri et al. [130] examined different MOFs for toluene adsorption under standard temperature and pressure conditions. The equilibrium toluene adsorption capacity on MOFs was changing in the following order: ZIF-67 (224 mg g^−1^) > UiO-66 (166 mg g^−1^) > MOF-199 (159 mg g^−1^) > MIL-101 (98.3 mg g^−1^). The highest adsorption capacity of ZIF-67 was attributed to its highest SSA of 1401 m^2^ g^−1^ among the MOFs studied. Xian et al. [44] determined the adsorption capacity of MIL-101 for 1,2-dichloroethane (9.71 mmol g^−1^), ethyl acetate (5.79 mmol g^−1^), and benzene (3.76 mmol g^−1^). However, the adsorption properties of MIL-101 decreased significantly in the presence of moisture due to competitive adsorption between water molecules and VOCs. Zhu et al. [131] synthesized a hydrophobic MIL(Cr)-Z1 with a high SSA 2080 m^2^ g^−1^ and large pore volume 1.23 cm^3^ g^−1^ using naphthalene dicarboxylic acid as a ligand. The benzene adsorption capacity of MIL(Cr)-Z1 at 20 °C and relative humidity of 5, 40, and 60% was 261.7, 229.6, and 205.4 mg g^−1^, respectively. Shafiei et al. [129] synthesized a modified MIL-101(Cr) using a different linker-to-cluster molar ratio (2:1 instead of 1:1) and HF and HNO_3_ as modifying agents for VOC adsorption. The resulting MOF possessed a very high SSA of 4293 m^2^ g^−1^, pore volume of 2.43 cm^3^ g^−1^, and high adsorption of n-pentane, n-hexane, n-heptane, benzene, toluene, and xylenes. The regeneration efficiency of modified MIL-101(Cr) (99.7%) was higher than that of commercial AC (87.2%).

**Figure 10:**
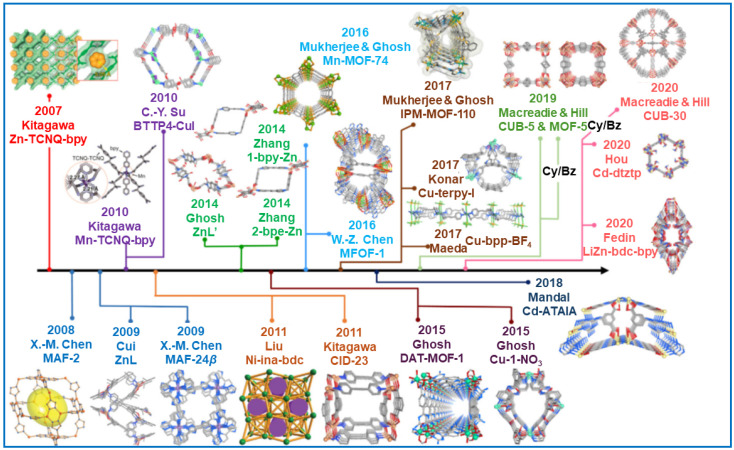
Chronology of discoveries in the design of MOFs that exhibit benzene (Bz) or cyclohexane (Cy) selectivity [132]. Reproduced with permission from ref. [132]. Copyright © 2021, Elsevier.

The authors of the review paper [132] presented the progress in the design of MOF adsorbents for selective adsorption and separation of benzene and cyclohexane and analyzed the key factors that govern the adsorption processes. Most of the analyzed MOFs show high adsorption selectivity for benzene over cyclohexane. It was concluded that the aromaticity of ligands is important to provide binding adsorption sites for benzene. However, for several MOFs, π···π interactions play a minor role (if any) in binding benzene molecules. Instead, it was revealed that CH···π and O···H and N···H interactions were essential in benzene binding. Figure 10 shows the chronology of the discoveries reported so far related to the selective benzene and cyclohexane adsorption [132].

Jhung et al. [133] synthesized MIL-101 with a high SSA of 3900 m^2^ g^−1^ for benzene adsorption. Its benzene adsorption capacity at 30 °C and under relative pressure P/P_0_ = 0.5 was as high as 16.7 mmol g^–1^. They compared this result with other porous materials such as mesoporous silica SBA-15 (SSA of 805 m^2^ g^–1^), HZSM-5 zeolite (SSA of 550 m^2^ g^–1^), and a commercial activated carbon (SSA of 1600 m^2^ g^–1^), for which the measured adsorption capacities at the same conditions were significantly lower, namely 3.0, 1.9, and 8.0 mmol g^–1^, respectively. The exceptional adsorption properties of the MOF were assigned to high porosity with relatively large pore sizes, which is beneficial for sufficient diffusion of benzene molecules within the internal sorbent structure. Additionally, small-sized MIL-101 nanoparticles might provide additional adsorption sites for electronegative, nonpolar benzene molecules.

MOFs usually exhibit an open structure with large void spaces and a relatively low contribution of unsaturated metal centers, thus often providing rather weak dispersion forces to effectively capture low-molecular-weight VOCs [134]. To overcome these difficulties, MOFs can be coupled with other porous materials that have a dense arrangement of atoms. Various MOF-based composites such as MOF-carbon, MOF-metal oxide, MOF-silica, and MOF-organic polymer have been developed as effective adsorbents for air purification [135]. Some additives may effectively increase porosity, dispersion forces, and the number of active sites in the MOF structure. Zheng et al. [136] reported that the adsorption capacity MIL-101/GO towards carbon tetrachloride was as high as 2368.1 mg g^−1^ at 30 °C, which was an increase of 16% compared to pure MIL-101 (2044.4 mg g^−1^). The adsorption capacity of MIL-101/GO significantly outperformed that of conventional adsorbents, such as activated carbon (600 mg g^−1^) or zeolite (430 mg g^−1^), implying their suitability for application in the field of chlorinated volatile organic compound (Cl-VOC) adsorption.

Apparently, MOFs are promising adsorbents for VOC removal because of their tunable pore structure, composition, and susceptibility to modifications and, therefore, adjustable physicochemical properties. In general, the VOC adsorption capacities of MOFs are often higher than those of conventional adsorbents such as ACs and zeolites. Nevertheless, there are still limitations hindering their industrial application, mainly associated with the high costs of organic linkers, synthesis complexity, and often poor reproducibility, as well as lower thermal and chemical stability in comparison to zeolites or ACs [137,138]. Moreover, due to large void spaces in MOFs, they usually offer poor dispersion forces for the physical adsorption of VOCs [135,139].

**Figure 11:**
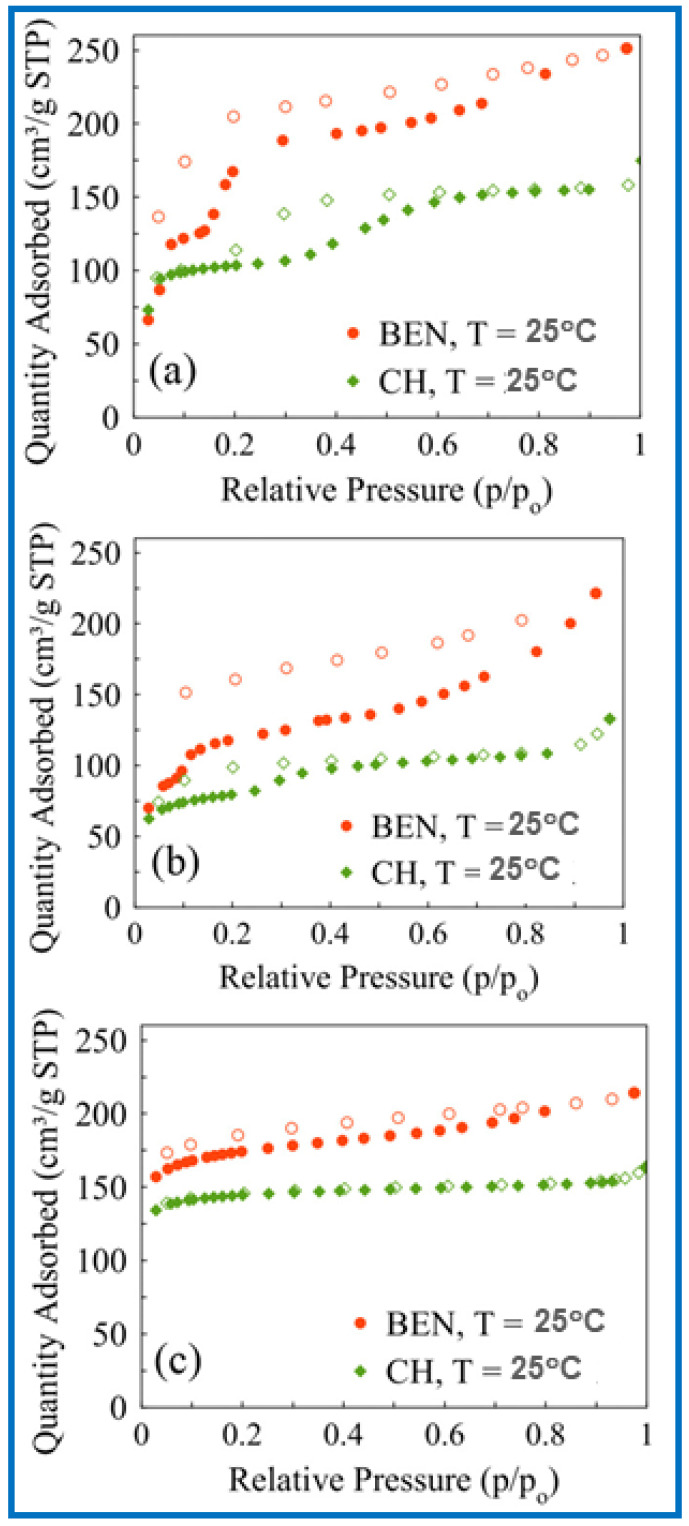
Benzene and cyclohexane adsorption isotherms acquired at 25 °C on (**a**) COF-300-rt (the syntheses at room temperature or in solvothermal conditions enabled to selectively isolate the narrow-pore form of COF-300), (**b**) COF-300-st (a mixture of the narrow-pore and a larger-pore form), and (**c**) LZU-111 (microporous framework synthesized at room temperature). Empty symbols denote a desorption branch [146]. Figure reproduced with permission from ref. [146]; licensed under CC-BY 4.0.

Covalent organic structures (COFs) are open structures formed by the condensation of organic monomers using covalent bonds. Since their introduction in 2005 by Yaghi et al. [140], intensive research has been devoted to the modular design and construction of COFs with desirable structure, composition, and physicochemical properties. Nowadays, there is a large variety of COFs with periodic, homogenous porosity with tunable pore size and functionalities of their walls. They are proposed for diverse applications, ranging from separation [141] to heterogeneous catalysis [142], environmental remediation [143], and electro-optical processes [144]. For instance, imine-based COFs show several advantages over other COFs, such as higher chemical stability and room-temperature synthesis [145]. Moroni et al. [146] investigated the influence of pore flexibility of imine-based COFs (COF-300 and LZU-111) on the adsorption and separation of benzene and cyclohexane. Optimized syntheses at room temperature or under solvothermal conditions enabled the selective isolation of COF-300 forms with narrow pores (COF-300-rt) and forms with mixed narrow and larger pores (COF-300-st), having different pore structure parameters SSA of 39 and 1270 m^2^ g^−1^, respectively. In the case of LZU-111, only room-temperature synthesis was successful, leading to a microporous framework. These three COF-300-rt, COF-300-st, and LZU-111 were examined for adsorption and separation of benzene and cyclohexane under static and dynamic conditions. All COFs showed a higher static adsorption capacity at 25 °C and a pressure of 1 bar toward benzene (251, 221, and 214 cm^3^ g^−1^ STP), respectively, compared to cyclohexane (175, 133, and 164 cm^3^ g^−1^ STP, respectively). Benzene and cyclohexane adsorption isotherms measured on COF-300 showed step-wise increasing adsorption, which is characteristic of porous materials with flexible pores. Figure 11 shows the adsorption and desorption isotherms of benzene and cyclohexane at 25 °C on the COFs studied. The suitability for selective benzene adsorption over cyclohexane of COFs was assessed using a 50:50 *v*/*v* benzene/cyclohexane flow at different temperatures (T = 25, 50, and 75 °C). LZU-111 did not selectively retain any of the two components, while COF-300 showed stronger benzene–COF interactions under dynamic conditions.

### 5.6. Zeolites

Zeolites are crystalline porous aluminosilicates consisting of a three-dimensional (3D) arrangement of TO_4_ tetrahedra (T is Al or Si) [147]. Zeolites are conventional microporous adsorbents characterized by a high SSA (250–800 m^2^ g^−1^), controlled microporosity, hydrophobicity, good thermal and chemical stability, and nonflammability. Different zeolite types have been proposed for the adsorption of VOCs, such as silicalite-1 (MFI-structure type), beta (*BEA-structure type), SSZ-23 (STT-structure type), and chabazite (CHA-structure type) [148,149,150]. Considering the industrial applicability of zeolites for VOC adsorption, one should keep in mind that under humid conditions, the influence of competitive water adsorption may significantly reduce their VOC adsorption capacity [151]. However, the properties of zeolites can be tailored, e.g., by changing the Si/Al ratio [152]; the Si content in zeolites is related to their water resistance, which can be adjusted during the synthesis process. For instance, adsorption of nonpolar VOCs (e.g., butyl acetate) in the presence of moisture on zeolites increases with increasing Si/Al ratio, which is attributed to the increase in hydrophobicity of the zeolite surface [153]. Zeolites such as MFI (ZSM-5) and FAU (NaX and NaY) with different Si/Al ratios were synthesized for the adsorption-based removal of dichloromethane vapors (at 30 °C and 5000 ppm) [149]. It was shown that ZSM-5, with the highest Si/Al ratio of 204.5, exhibited the highest dichloromethane adsorption capacity of 179.2 mg g^−1^ and was not as susceptible to a relative humidity of 10–90%. Lee et al. [154] studied the adsorption and thermal desorption of acetone and toluene vapors on a dealuminated Y-zeolite at 20 °C, 4500 ppm, and in an N_2_ atmosphere. The results indicated that zeolite Y with an SSA of 704 m^2^ g^−1^ and pore volume of 0.47 cm^3^ g^−1^ can be used in cycling the adsorption–desorption of VOCs without a significant decrease in its adsorption capacity after several regeneration cycles. A similar conclusion was also reported in the case of NaY zeolite for cycling n-hexane (500 ppm) adsorption [155]. Another approach to increase the selectivity in VOC adsorption on zeolites is creating proper zeolite-based composites, including core–shell nanostructures [151]. For instance, the core–shell architecture constructed of ZSM-5 framework and LDH-based shell afforded efficient and selective adsorption of VOCs under high humidity [151]. The LDH shell provided increased hydrophobicity and enhanced m-xylene adsorption capacity of the composite in comparison to ZSM-5 zeolite.

Depositing zeolite crystals on the surface of macro- or mesoporous supports, e.g., clay minerals, MOFs, ordered mesoporous silicas, etc., can be an effective strategy to overcome low diffusion and mass transport within solely microporous zeolite crystals. For instance, diatomite (Dt) was proposed as a macroporous support (pore size 50–800 nm) to prepare Dt-zeolite composites [156,157]. The benzene adsorption capacity of hierarchically porous Dt/MFI-type zeolite composites of 62.5 mg g^−1^ at 25 °C was higher than that of Dt. The Dt/silicate composite showed a high benzene adsorption capacity of 246.0 mg g^−1^ at 25 °C, which was much higher than that of Dt and silicate [158] due to improved dispersion and reduced mass transfer resistance.

Zeolites usually show the lower adsorption capacity of VOCs compared to ACs, MOFs, or other sorbents with high porosity [159]. However, thanks to their uniform micropores with controlled sizes and tailorable water content, they may exhibit good VOC adsorption selectivity [55]. These features of zeolites are used in the preparation of zeolite-based catalysts (e.g., with catalytically active metals) for the effective catalytic oxidation of VOCs, often better than that of conventional catalysts on oxide supports [160,161].

Zeolites’ advantages over ACs involve their excellent hydrothermal and chemical stability, nonflammability, and lower temperatures needed for desorption [162]. However, these sorbents possess intrinsic disadvantages in terms of VOC adsorption, which results from monomodal microporosity (pores below 2 nm) and nanoparticle agglomeration, which hinder the diffusion and mass transfer of larger adsorbate molecules, limiting their adsorption properties [163]. Moreover, their synthesis process often involves expensive precursors such as tetraethyl orthosilicate and cetyltrimethylammonium bromide, especially in comparison to raw materials used as precursors for AC synthesis [164].

### 5.7. Siliceous Adsorbents

Ordered mesoporous inorganic solids have attracted great attention since the first synthesis of ordered mesoporous silicas (OMSs) by Kresge et al. [165] in 1992. The pore size in these molecular sieves, as well as the chemical composition of the wall surface, can be altered by using different organic surfactants (cationic, anionic, and non-ionic) as templates. The first reported mesoporous silica was synthesized using surfactant templates at the Mobil Research and Development Corporation laboratory and was denoted as MCM-41 (MCM, Mobil Composition of Matter). Another type of OMS was synthesized by using block copolymers as templates; the representative silica, denoted as SBA-15 (SBA, Santa Barbara Amorphous), was first synthesized at the University of California in Santa Barbara [166]. Both silicas exhibit a hexagonal pore order, but SBA-15 shows higher stability, thicker walls, and larger pore volume compared to MCM-41. Moreover, SBA-15 possesses disordered micropores inside the silica walls [167].

**Figure 12:**
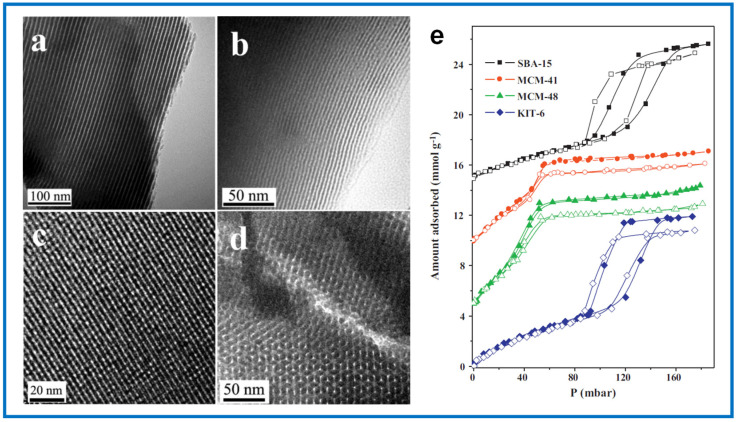
Transmission electron microscopy (TEM) images of the phenyl-grafted (**a**) SBA-15, (**b**) MCM-41, (**c**) MCM-48, and (**d**) KIT-6. (**e**) Adsorption isotherms of benzene on pure (filled symbols) and phenyl-grafted (open symbols) SBA-15, MCM-41, MCM-48, and KIT-6 at 35 °C. The isotherms for the original and phenyl-grafted MCM-48, MCM-41, and SBA-15 were shifted by 5, 10, and 15 mmol g^−1^, respectively [168]. Reproduced with permission from ref. [168]. Copyright © 2011, Elsevier.

The static adsorption of benzene and the dynamic adsorption of benzene and a bicomponent mixture of benzene and cyclohexane were evaluated on silicas with different pore structures, including SBA-15, MCM-41, MCM-48, and KIT-6 and functionalized silicas. [168]. Surface functionalization with phenyltriethoxysilane by a post-synthesis grafting approach afforded silicas with covalently anchored phenyl groups and the retained long-range order of their mesoporous channels. This functionalization was essential to increase the hydrophobicity and affinity for aromatic compounds compared to pure silicas. The pore structure and surface chemistry significantly influenced their VOC adsorption behavior. The best adsorption performance in single- and bi-component dynamic adsorption showed phenyl-grafted KIT-6 silica because of its relatively large mesopores and bicontinuous cubic pore structure, which provided good accessibility for functionalization, leading to the highest amount of the anchored surface functional groups among silicas studied. The lowest adsorption efficiency was observed for the functionalized MCM-41, probably due to the small mesopore size and 1D mesoporous channels of MCM-41, unfavorable from the viewpoint of functionalization due to the hindered access to the internal pore surface. In the static adsorption experiment, the equilibrium adsorption capacities of benzene on phenyl-grafted silicas were lower than those on the pure silicas, which corresponded to a reduction in the pore volume upon functionalization. Among the silicas studied, the highest equilibrium adsorption capacity at 35 °C of 11.93 mmol g^−1^ showed KIT-6 because of its highest total pore volume of 1.29 cm^3^ g^−1^. After functionalization, its adsorption capacity dropped to 10.71 mmol g^−1^ (Figure 12). The adsorption capacity of MCM-41 and SBA-15 under the same conditions were 7.28 and 10.64 mmol g^−1^, respectively.

Three mesoporous silicas were synthesized using different post-synthetic treatments of MCM-41-type OMS: pure MCM-41, pure MCM-41 with expanded pores, and surfactant-containing MCM-41 with expanded pores and examined for adsorption of chlorinated and aromatic hydrocarbons [169]. The surfactant-containing silica showed good compatibility with chlorinated compounds in adsorption experiments from gaseous streams containing moisture. The pure examined silicas showed higher selectivity and adsorption capacity towards aromatic hydrocarbons. Among the samples studied, the highest benzene adsorption capacity of 2.43 mmol g^−1^ at a relative pressure of P/P_0_ = 0.10 exhibited pure MCM-41, which was attributed to the strong interactions between the silica surface and the π-electrons of aromatic hydrocarbons. Elsewhere [170], the adsorption of benzene, toluene, and phenol was investigated using different sorbents, i.e., MCM-41 silica and three organo-zeolites. The highest sorption capacity towards all VOCs studied showed the synthesized MCM-41. However, in this paper, good adsorption properties towards VOCs of organo-natural clinoptilolite (surfactant-modified zeolite) were also highlighted, especially considering the significantly lower costs of its preparation in comparison to mesoporous silicas. Greater insight into the mechanisms of VOC adsorption on OMSs is needed to design modified silicas with a superior VOC adsorption capacity.

The advantages and disadvantages of different VOC adsorbents and types of applicable VOCs are summarized in Table 3.

## 6. Conclusions and Perspectives

Emissions of VOCs, including benzene, increase dramatically with accelerated industrialization and economic growth. Most VOCs can lead to environmental pollution and pose a threat to human health due to their toxic and carcinogenic nature. It is urgent to look for efficient and sustainable ways to purify air and water from VOCs. Adsorption is considered one of the most promising technologies for adsorbing VOCs due to its cost-effectiveness, operational flexibility, and low energy consumption. In particular, adsorption-based technologies have great potential to capture valuable aromatic VOCs from industrial exhaust gases and recycle both adsorbates and adsorbents.

Porous materials, such as carbons, zeolites, silicas, metal-organic frameworks, and covalent organic frameworks, are widely proposed for the adsorption of VOCs, and have been reviewed in this paper. Research on these materials is focused on their adsorption capacity, hydrophobic/hydrophilic properties, stability, and regeneration ability. In this review, the possibilities of VOC adsorption on different groups of porous materials, such as activated carbons, active carbon fibers, ordered mesoporous carbons, graphene-based materials, metal-organic frameworks, covalent organic frameworks, zeolites, and silicas, are summarized. It seems that among these materials, great attention has been paid to porous carbon-based materials and MOFs because of the ease of adjusting their surface chemistry and structural parameters for targeted VOC adsorption, leading to superior adsorption properties. The key factors influencing the adsorption of VOCs include specific surface area, pore volume, pore size, chemical functional groups of adsorbent, molecular size, polarity, boiling point of adsorbate, and adsorption conditions, including adsorbate concentration, temperature, and humidity.

It is still debatable which factor is dominant in the adsorption of VOCs: porosity or surface chemistry. To find the answer, the adsorption behavior of VOCs on a series of porous adsorbents should be comprehensively investigated. Apparently, the mechanisms of adsorption are still insufficiently known, especially those involving interactions between functional groups of the adsorbent and VOC molecules. Based on the reviewed examples and results, it can be summarized that both the structure parameters and chemical functional groups attached to the adsorbent surface highly influence VOC adsorption. Physical and chemical modifications, e.g., thermal treatment, acid/alkaline treatment, heteroatom doping, metal/metal oxide decoration, organic polymer coating, radiation, etc., can be used to alter both the pore structure and surface chemistry of adsorbents. The changes are usually directed to create additional micro- or mesopores and introduce new (or replace) functional groups. In general, high porosity with a large contribution of narrow pores (micropores) facilitates adsorption, and the influence of functional groups is related to the polarity of VOCs. The physicochemical properties of VOCs have a major influence on their adsorption. The diffusion of large VOC molecules into small pores is sterically hindered; therefore, the adsorption capacity of solely microporous sorbents is negatively correlated to molecular cross-sectional areas of VOCs [15].

The polarity of VOCs is an important factor, and their adsorption can be altered by proper modifications of the adsorbent’s surface, e.g., the incorporation of acidic or basic surface functional groups. Strong adsorption interactions are observed for VOCs with high boiling points. However, their desorption is more difficult than in the case of VOCs with lower boiling points. Adsorption conditions such as temperature and water vapor content have a significant impact on the adsorption of VOCs. Low temperature favors the adsorption of VOCs because the process usually involves physical adsorption, which is an exothermic process. In general, the presence of water vapor reduces the adsorption capacity of sorbents toward VOCs due to the competitive adsorption of water and VOC molecules.

Although significant progress has been made in the field of VOC adsorption on porous materials, there are still limitations and uncertainties that need to be clarified. Additional research is still needed to (a) increase the adsorption rate of VOCs on porous materials, (b) reduce production costs of efficient VOC sorbents or find alternatives, (c) increase the adsorption of low-boiling-point VOCs, (d) solve the problem of hindered desorption of high-boiling-point VOCs, (e) increase the selectivity of VOC adsorption, including selective adsorption in the presence of water vapor, (f) improve the recoverability of VOC sorbents, assuring the long life of cycling adsorption–desorption of VOCs, and (g) ensure the proper disposal of used adsorbents to avoid secondary environmental pollution and threats to human health. Research dealing with the adsorption of benzene and other VOCs on porous adsorbents still faces many challenges.

New technologies hold great potential for improving the removal of VOCs from various processes, thereby reducing their environmental impact. Technologies that facilitate the development of manufacturing methods, advanced filtration systems, catalytic converters, or new adsorbents that could effectively capture VOCs from flue gases are critical in achieving these goals. Innovations in this field are highly desirable, as reducing costs and enhancing the efficiency of VOC removal systems make them more accessible and effective across a wide range of industries. In addition, advancements in the synthesis of inexpensive, efficient, and selective sorbents of VOCs are needed. Specifically, developing synthesis procedures that enable the production of desired sorbents utilizing green chemistry principles, e.g., from cheap precursors with low energy consumption and minimal amounts of generated by-products and wastes, would greatly enhance the process sustainability and profitability. Additionally, the scalability of synthesis processes is another critical factor in achieving these objectives. Overall, leveraging emerging technologies, refining chemical processes, and addressing scalability challenges can lead to more efficient, cost-effective, and environmentally friendly methods for VOC removal and their broader industrial applications.

## Figures and Tables

**Table 1:** Classification and characterization of volatile organic compounds (VOCs) [19]. Reproduced with permission from Ref. [19]. Copyright © 2021, Elsevier.

Category	VOCs	Relative Molecular Mass	Boiling Point (°C)	Kinetic Diameter (nm)
Saturated alkanes	Cyclohexane	84.16	80.7	0.60
	n-Hexane	86.18	69	0.43
	Ethane	30.07	−88.6	0.44
	Propane	44.09	−42.09	0.43
Unsaturated alkenes and alkynes	Ethylene	28.06	−104	0.41
	Propene	42.08	−47.4	0.46
	Butene	56.10	−6.90	0.45
	Acetylene	26.04	−83.8	0.33
Aromatic hydrocarbons	Benzene	78.11	80.1	0.59
	Toluene	92.14	110.6	0.53
	Xylene	106.16	144.4	0.68
	Naphthalene	128.18	217.9	-
	Ethylbenzene	106.16	136.2	0.58
Oxygen-containing VOCs	Methanol	32.04	64.7	0.36
	Ethanol	46.07	78.3	0.45
	Acetone	58.08	56.53	0.46
	Ethyl acetate	88.11	77	0.50
Chlorinated VOCs	Dichloromethane	84.93	39.75	0.49
	1,2-Dichloroethane	98.96	83.5	-
	Trichloroethylene	131.39	87.1	0.73
	Chlorobenzene	112.56	132.2	-

**Table 2:** Summary of the adsorption capacities of activated carbons (ACs) for different volatile organic compounds (VOCs) [15]. Reproduced with permission from ref. [15]. Copyright © 2017, Elsevier.

Adsorbate	Formula	Formula Weight (g mol^−1^)	Adsorption Capacity (mg g^−1^)	Conditions
Acetone	C_3_H_6_O	58.08	483.09	25 °C, 800 L h^−1^
Acetone	C_3_H_6_O	58.08	343.89	20 °C, 5 L h^−1^, 50 g m^−3^
Benzene	C_6_H_6_	78.11	27.50	25 °C, 3.6 L h^−1^, 6000 ppm
Benzene	C_6_H_6_	78.11	161.42	0.680 P_0_
Butanol	C_4_H_9_OH	74.12	262.38	20 °C, 625 Pa
Butanone	C_4_H_8_O	72.11	24.30	25 °C, 60 mL min^−1^, 6000 ppm
Butanone	C_4_H_8_O	72.11	364.88	20 °C, 5 L h^−1^, 50 g m^−3^
Cyclohexane	C_6_H_12_	84.16	327.18	25 °C, <0.2 P_0_
Dichloromethane	CH_2_Cl_2_	84.93	360.95	30 °C, 742 Pa, 5 mL min^−1^
Ethanol	C_2_H_6_O	46.07	15.90	25 °C, 60 mL min^−1^, 6000 ppm
Ethanol	C_2_H_6_O	46.07	389.84	20 °C, 5 L h^−1^, 50 g m^−3^
Ethyl acetate	C_4_H_8_O_2_	88.11	420.92	25 °C, 0.740 P_0_, 6000 ppmv
Ethyl acetate	C_4_H_8_O_2_	88.11	450.24	20 °C, 9686 Pa
Ethyl acetate	C_4_H_8_O_2_	88.11	388.65	20 °C, 5 L h^−1^, 50 g m^−3^
Isopropyl acetate	C_5_H_10_O_2_	102.13	147.45	25 °C, 0.822 P_0_
Isobutyl acetate	C_6_H_12_O_2_	116.16	151.71	25 °C, 0.7222 P_0_
Methanol	CH_3_OH	32.04	10.60	25 °C, 60 mL min^−1^, 6000 ppmv
Methyl acetate	C_3_H_6_O_2_	74.08	165.54	25 °C, 0.497 P_0_
m-Xylene	C_8_H_10_	106.16	31.09	0.606 P_0_
m-Xylene	C_8_H_10_	106.16	292.40	25 °C, <0.2 P_0_
n-Hexane	C_6_H_14_	86.18	379.90	25 °C, <0.2 P_0_
n-Propanol	C_3_H_8_O	60.10	30.30	25 °C, 60 mL min^−1^, 6000 ppm
n-Propyl acetate	C_5_H_10_O_2_	102.13	199.64	25 °C, 0.5053 P_0_
n-Butyl acetate	C_6_H_12_O_2_	116.16	260.80	25 °C, 0.6154 P_0_
o-Xylene	C_8_H_10_	106.16	305.70	22–27 °C, 45 mL min^−1^, 2176–2239 mg m^−3^
o-Xylene	C_8_H_10_	106.16	90.40	25 °C, 60 mL min^−1^, 6000 ppmv
o-Xylene	C_8_H_10_	106.16	111.47	0.741 P_0_
p-Xylene	C_8_H_10_	106.16	29.46	0.556 P_0_
Toluene	C_7_H_8_	92.14	364.96	25 °C, 0.8 m^3^ h^−1^
Toluene	C_7_H_8_	92.14	59.20	25 °C, 60 mL min^−1^, 6000 ppmv
Toluene	C_7_H_8_	92.14	109.45	0.818 P_0_
Toluene	C_7_H_8_	92.14	366.72	20 °C, 2910 Pa
Toluene	C_7_H_8_	92.14	424.40	20 °C, 5 L h^−1^, 50 g m^−3^
1,1,1-Trichloroethane	C_2_HO_2_Cl_3_	163.40	765.60	25 °C, <0.2 P_0_
1,2-Dichloroethane	C_2_H_4_Cl_2_	98.96	377.22	25 °C, 0.8 m^3^ h^−1^
1,2-Dichloroethane	C_2_H_4_Cl_2_	98.96	526.67	20 °C, 5 L h^−1^, 50 g m^−3^
2-Ethyl-4-methyl-1,3-dioxolane	C_6_H_12_O_2_	116.16	415.61	20 °C, 5 L h^−1^, 50 g m^−3^

**Table 3:** Advantages and disadvantages of different VOC (volatile organic compound) adsorbents and types of applicable VOCs [87]; license under CC-BY.

Sorbent	Prons	Cons	Applicable VOC Types
Raw materials	Environmentally friendly, acid and alkali resistance, low cost	Rather low porosity and adsorption capacity, incomplete adsorption/desorption, difficult regeneration	Small molecular weight, medium to high polarity, gaseous, water-soluble, and aromatic VOCs
Activated carbon/biochar	Environmentally friendly, moderately efficient adsorption, safety	Slow adsorption speed, poor adsorption selectivity	Larger molecular weight, medium to low polarity, and oxygenated VOCs
Activated carbon fiber	Fast adsorption, easy regeneration, high adsorption capacity	High cost, easy to saturate, poor specificity	Small molecular weight, low to medium polarity, and aromatic groups containing VOCs
Graphene-based materials	Highly efficient adsorption, high stability, renewability	Slow adsorption speed, easy to saturate, can only be used in specific environments, high cost	Small molecular weight, polar, and aromatic VOCs
MOFs	High adsorption capacity, their framework structure is predictable, versatile, and tunable	High cost of organic precursor preparation, poor thermal stability	A variety of VOCs with different molecular weights, polarities, and chemical properties
Zeolites	Adjustable pore size, uniform microporous structure, rich framework diversity, selective adsorption according to the molecular size of VOCs, thermal stability, easy to regenerate	Adsorption is affected by temperature and humidity, the relatively high cost of precursors for synthetic zeolites	Small molecular weight, low polarity, for VOCs that are stable at moderate to high temperatures.
Ordered mesoporous materials	Adjustable pore size and easy surface functionalization, uniform mesoporous structure, rich framework diversity, selective adsorption according to the molecular size of VOCs, facilitated diffusion	High-cost, multistep preparation	Large molecular weight, a variety of VOCs with different polarities, and chemical properties

## Data Availability

Not applicable.
